# Reversible Conjugation of Polypeptides and Proteins Utilizing a [3.3.1] Scaffold under Mild Conditions

**DOI:** 10.1021/acs.orglett.4c02228

**Published:** 2024-07-22

**Authors:** Ryan J. Bartlett, Kelly D. Crisostomo, Qiang Zhang

**Affiliations:** Department of Chemistry, University at Albany, State University of New York, 1400 Washington Avenue, Albany, New York 12222, United States

## Abstract

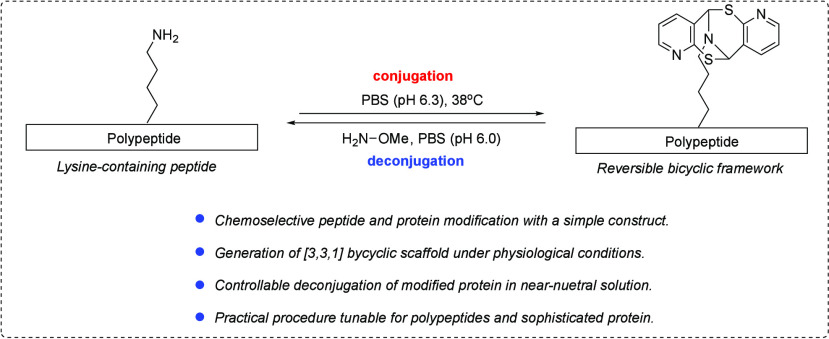

An investigation of reversible protein conjugation and deconjugation is presented. Despite numerous available protein conjugation methods, there has been limited documentation of achieving protein conjugation in a controlled and reversible manner. This report introduces a protocol that enables protein modification in a multicomponent fashion under aqueous buffer and mild conditions. A readily available mercaptobenzaldehyde derivative can modify the primary amine of peptides and proteins with a distinctive [3.3.1] scaffold. This modification can be reversed under mild conditions in a controlled fashion, restoring the original protein motif. The effectiveness of this approach has been demonstrated in the modification and quantifiable regeneration of insulin protein.

Post-translational modifications are responsible for the modulation, functionalization, and promotion of the physical properties of a peptide or protein.^1^ These modifications have spawned various research-driven applications that are already being utilized across a broad therapeutic market.^2^ The quest for more efficient and specific protein conjugation strategies continues, with a significant focus on the controlled, reversible formation of covalent bonds.^3^

Common methods toward reversible modification of a peptide backbone is through the use of electrophilic aldehydes and ketones, which are often chemoselective for lysine residues,^4^ although p*K*_a_^5^ differences between the side chain and the N-terminal (α-amino) amines have also been leveraged for alternative chemoselective strategies.^6^ The resulting imine motif is rapidly disintegrated via hydrolysis at physiological pH.^7^ To increase the stability of the conjugation, creative scaffolds are employed that stabilize imine via the formation of an additional bond.^8^ These tactics produce attachment via lysine in typically inapt conditions, and it is then possible to instigate detachment of the conjugate through disruption of an additional bond.^9^ This approach to reversibility has shown promising results in biological research,^10^ but the scarcity in available methods makes the development of mild, stimulus-driven deconjugation strategies an area of interest.^11^ To address this, we have investigated the deconjugation of several mercaptan scaffolds derived from the one-pot three-component protocol established by previous chemistry.^12^ Herein, we present a reversible method for the controlled, selective modification of amino groups in polypeptides and proteins.

The investigation began with screening mercaptan scaffolds for suitable formation of the bicyclic framework on a common peptide. Both aromatic and non-aromatic mercaptan aldehyde motifs were obtained from simple synthesis or commercially sourced. Dimerized benzaldehyde **3** and heterocycle **6** were synthesized from commercially available compounds **1** and **4** in two steps, and substituted mercaptan **9** was obtained via an alternative two-step route (Scheme 1).^13^ Non-aromatic, dimerized mercaptan aldehyde **10** and commercial di/monomer mixture **11** were directly purchased. Each design was subjected to peptide conjugation conditions in the presence of 14-mer peptide **12** in aqueous buffer (Table 1). We found that, in near neutral solution (pH 6.3), mercaptobenzaldehyde **3** and its derivative **6** converted to the [3.3.1] bicyclic products **13** and **14**, respectively. Diol **10** enjoyed substantial conversion as well and produced a [2.2.1] bicyclic frame conjugate on peptide **12**. There was no observed bicyclic formation for compound **11**/**11a** under the same conditions, likely as a result of the less reactive mercaptan ketone construct. Benzaldehyde derivative **9** was observed to achieve around 50% conversion to product **17** after 24 h, and extending the reaction to 48 h lead to the decomposition of the scaffold. Evaluation for a proof of concept resumed on the bicyclic scaffolds derived from compounds **3**, **6**, and **10**.

**Scheme 1:**
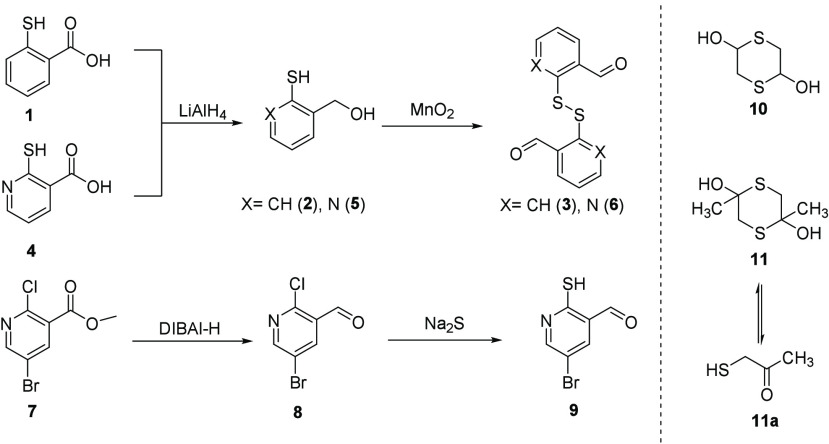
Synthesis of Mercaptan Conjugates

**Table 1:**
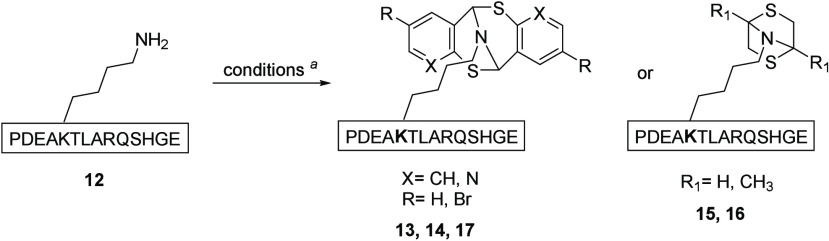
Screening of Bicylic Formation on Compound **12**

entry	X	R	R_1_	*t* (h)	yield (%)^b^
13	CH	H		24	53
14	N	H		48	91
15			H	24	33
16			CH_3_	24	0
17^c^	N	Br		48	0 (50)^d^

a Standard reaction conditions: compound **12** (0.0037 mmol), compounds **3**, **6**, **10**, and **11** (0.0148 mmol), and TCEP·HCl (0.0518 mmol) in PBS buffer (pH 6.3, 300 μL) at 38 °C until the starting peptide was no longer observed on LCMS.

b Isolated yields.

c A total of 0.0206 mmol of compound **9**.

d Observed conversion at 24 h, with decomposition upon formation.

Purifications of the conjugated product encountered issues. Initial attempts to purify compound **14** were unsuccessful through preparative high-performance liquid chromatography (HPLC). The liquid chromatography–mass spectrometry (LCMS) spectrum of purified compound **14** fractions suggested the cleavage of the conjugate during the HPLC purification, because decomposed material and the starting peptide were found in small quantities after the isolation. Although the conjugated product is stable in a neutral pH environment, we believe that the highly acidic additive [trifluoroacetic acid (TFA)] in the HPLC mobile phase facilitates the disintegration of compound **14**. Given the relative cleanliness of the crude product, low-pressure flash chromatography may be sufficient to isolate the peptide.^14^ The isolation issue of compound **14** was mitigated using a hand-packed C2 reverse-phase column with a H_2_O and MeCN mobile phase without any acid additive. The effect of the treated mobile phase was less significant on peptides **13** and **15** and was successfully isolated via HPLC.

The deconjugation of the modified peptides was investigated. Identifying a controllable practice for the removal of conjugates at a near neutral pH would be ideal. Recognizing that both [3.3.1] and [2.2.1] bicyclic frameworks contain N and S acetal carbons resembling thiazolidines, we hypothesized that invoking similar conditions to thiazolidine ring openings would restore the imine moiety,^15^ and the resulting hydrolysis would allow preconjugated peptides/proteins to be recovered. Deconjugation from peptide **14** was observed using LCMS after treatment with the water-soluble reagent methoxyamine–HCl (H_2_N–OMe·HCl and PBS at pH 6.0 for 21 h) in phosphate-buffered saline (PBS) buffer. Deconjugation of compounds **13** and **15** was unsuccessful under identical conditions. We did not observe regeneration of peptide **12** during deconjugation testing of the [2.2.1] bicyclic frame, and the deconjugation of compound **13** can be achieved under a much harsher environment (80 °C or pH 1).

With the combination of factors of conjugation yields and mild requirements of decoupling, compound **6** was employed as the scaffold for scope evaluation (Table 2). Each linear peptide was synthesized by solid-phase peptide synthesis (SPPS) and outfitted with random amino acid sequences, ensuring that the reaction tolerance of each residue was tested. All peptides were capped with proline on the N terminus to prevent binding to terminal amine and additionally to increase the efficacy of the C2 reverse-phase purification method.^16^

**Table 2:**
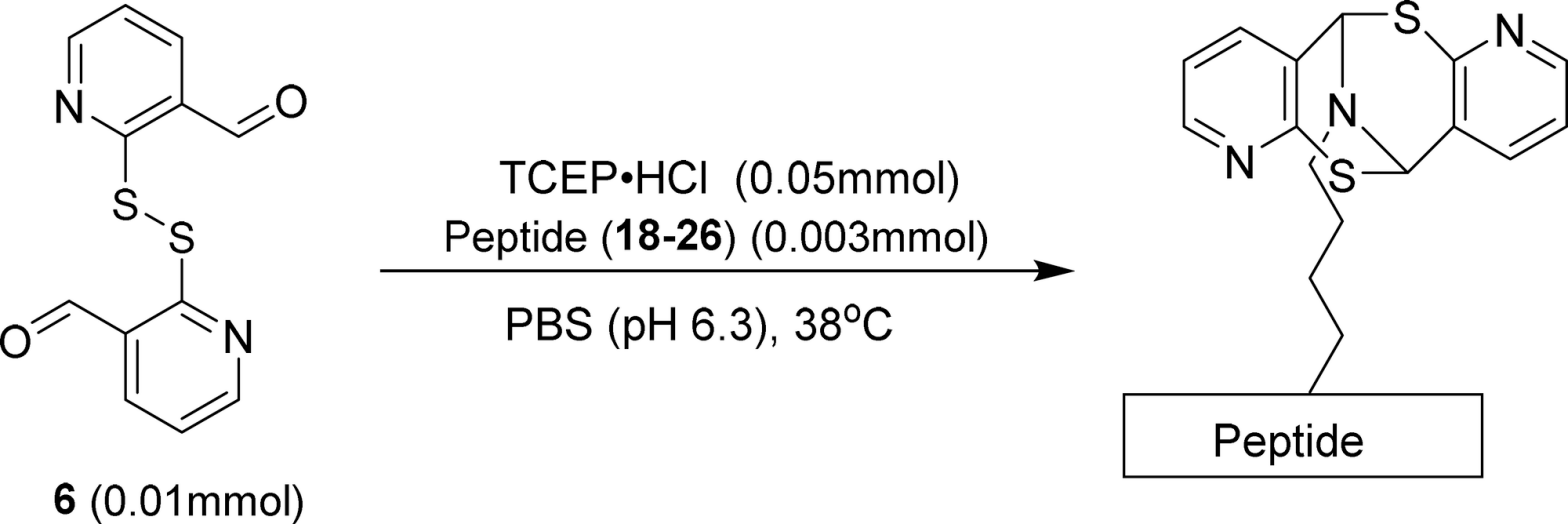
Investigation of the Compound **6** Conjugation Scope

entry	sequence	*t* (h)^a^	yield (%)^b^
27	PATRAG**K**ALNENLP	24	34^c^
28	PGLCAGA**K**LGP	24	0
29	PGL**K**AGAMLGP	38	84
30	PISRALL**K**AGTS	24	93
31	PG**K**GSWANALGT	18	55
32	PGEMSATQ**K**ARAREGF	21	16^d^
33	PGEMSATQ**K**AMAMEGF	48	0 (25)^e^
34	PL**K**AAQGIANP	18	68
35	PIAVGYLG**K**TAP	27	42^d^

a Time until conversion was completed, observed by LCMS.

b Isolated yield.

c Observed completed reaction from the starting peptide, with purification afforded by many fractions combined with the buffer salt runoff.

d Observed completed reaction from the starting peptide, with purifications hampered by less soluble groups.

e Conversion after 48 h, with the inability to isolate.

As evaluations concluded, most amino acids allowed for selective modification to lysine with high conversion and led to a single bicyclic [3.3.1] nonane conjugation product. This comes with the exception of peptides containing free cysteine, as with compound **28** as an example. Free thiol involves itself in the conjugation, and a myriad of products are observed on LCMS. Solubility and the efficiency of C2 column purification were the two main reasons attributed to lower isolation yields of some sequences. The sequences (e.g., compounds **32** and **33**) that are more hydrophobic tend to dissolve poorly in mobile eluents, resulting in low isolation yields. As a result of the limited efficiency of manual flash C2 purification under house air pressure, some fractions contained both buffer salt and product, leading to incomplete separation. Consequently, we chose to report yields based only on completely isolated fractions, which resulted in lower isolated yields (e.g., compound **27**). Peptides that avoid these two detriments achieve excellent yields (compounds **29** and **30**).

Subsequent deconjugation from peptides was attempted on modified sequences using H_2_N–OMe·HCl [PBS buffer at pH 6.0 and from room temperature (rt) to 38 °C for 6–21 h]. It quickly became clear that the concentration of methoxyamine and the reaction temperature were two key factors for deconjugation, which varied with different peptide sequences (Table 3). The course of the experiment was adjusted to evaluate conditions required to ensure each deconjugation would proceed within a 24 h period. With the ensurance that the solution remained steady around pH 6.0, the conditions were modified until each conjugate was substantially removed from the peptide. The deconjugation of compound **14** was achieved again at room temperature with 26 mM methoxyamine in 21 h (as illustrated in HPLC traces), and peptide **12** was recovered with 85% yield. Deconjugation of compound **27** was achieved within 6 h by the same method. The identical conditions could not release the original peptide **21** and slightly elevating the temperature (38 °C), and mixing buffer with a higher concentration of methoxyamine (42 mM) furnished deconjugation of the scaffold from compound **30** (55%). The reaction of compound **34** achieved a 95% isolation yield with another increase in the methoxyamine concentration, whereas compound **35** yielded compound **26** in 6 h with relatively similar conditions after HPLC. The deconjugation to restore original peptides achieved high yields overall.

**Table 3:**
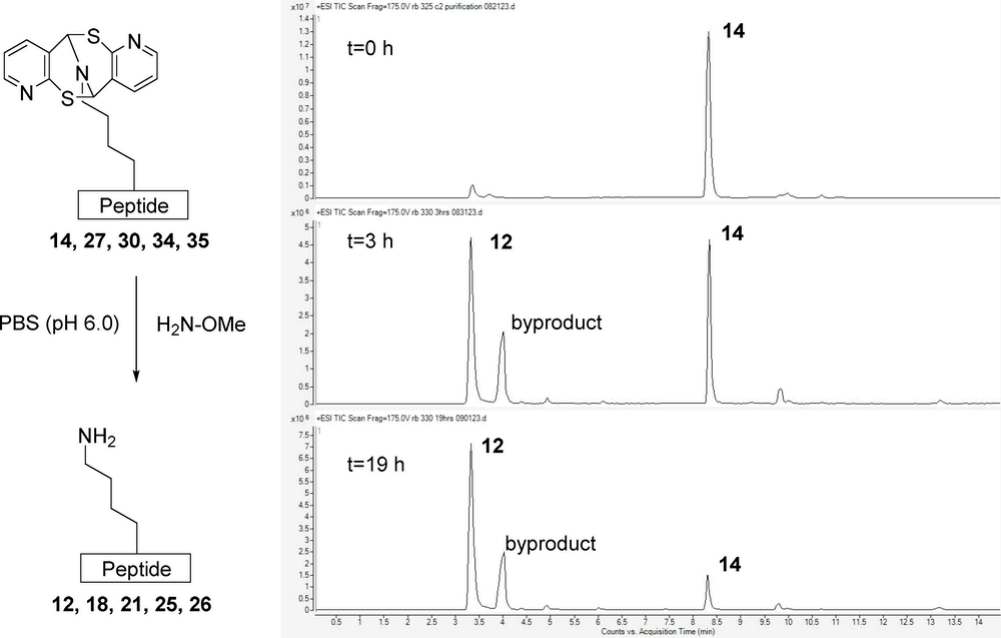
Investigation of the Deconjugation Scope

sequence	H_2_N–OMe (mM)	temperature (°C)	*t* (h)^a^	yield (%)^b^
PDEA**K**TLARQSHGE **14**	26	25	21	85
PATRAG**K**ALNENLP **27**	26	25	6	93
PISRALL**K**AGTS **30**	26	25	24	NR^c^
PISRALL**K**AGTS **30**	42	38	20	55
PL**K**AAQGIANP **34**	48	38	27	inc.^d^
PL**K**AAQGIANP **34**	76	38	21	95
PIAVGYLG**K**TAP **35**	14	38	36	NR^c^
PIAVGYLG**K**TAP **35**	67	38	6	86

a Time taken until substantial deconjugation was observed on LCMS.

b Isolated yields.

c No conversion.

d Incomplete deconjugation.

We next illustrate the practicality of controllable protein/peptide conjugation and deconjugation. Recombinant insulin was deemed sophisticated enough to test how readily the bicyclic framework derived from conjugation with compound **6** could be degraded. With unquestionable biological importance, insulin is still a relevant candidate for the adoption of additional therapeutic properties via modification.^17^ Insulin also enjoys several disulfide connections capping each cysteine; a well-mannered reaction would allow the biheterocyclic scaffold to form without the aforementioned thiol side-chain interference. Dimeric compound **6** could not be reduced *in situ* because TCEP·HCl would render undesired denaturing of insulin. Alternatively, dimer **6** was reduced to monomeric compound **6a** with TCEP·HCl prior to the conjugation. The monomer was then added to the buffer solution containing insulin after removal of the reagent by thoroughly washing with water (Figure 1c).

**Figure 1:**
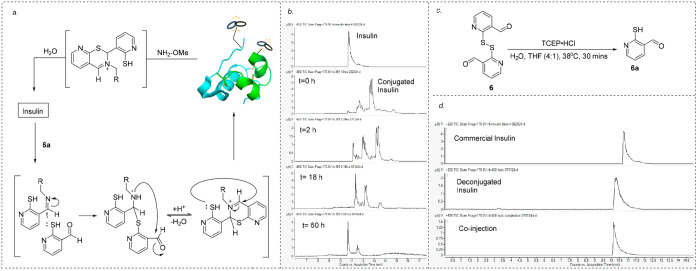
(a) Conjugation and deconjugation of insulin. (b) Raw electrospray ionization (ESI) trace of the deconjugation of insulin was obtained over 60 h. (c) Reduction of compound **6** to compound **6a**. (d) Co-injection of commercial insulin and deconjugated insulin.

Initial attempts at conjugation were stalled midway by the low solubility of insulin in the aqueous solution. Employing a 4:1 ratio of buffer and tetrahydrofuran (THF) dissolved the protein, allowing for complete conversion at pH 6.3 within 18 h. At near-neutral pH, the primary amine of a protein remains largely cationic, minimizing the differentiation between the N terminals and lysine side chains.^18^ This is observed when insulin was modified with compound **6a**, where two partially overlapped products were detected in LCMS (Figure 1b). Both products underwent double conjugation, and tandem mass spectrometry (MS/MS) revealed that at least one of the products features attachments on both lysine and the A-chain N terminus (Gly). Although it is expected to observe B-chain N-terminal modification at pH 6.3,^19^ corresponding mass was not found in either product peak. The observation might be attributed to the disintegration of the conjugate during the MS/MS analysis and failed to validate B-chain Phe modification.

The conjugation reaction solution was passed through a series of C8 plugs with no acid additive to remove the buffer salts.^20^ The filtrated insulin conjugate was subjected to subsequent deconjugation conditions, and we hypothesize that the N-terminal connections would not be detrimental to the reversible reaction (Figure 1a).^21^ LCMS monitoring of conjugated insulin in a solution of 7 mM competitive amine reagent was performed, and in 60 h, the conjugation protein peaks had disappeared, successfully replaced with the regenerated starting insulin without cleavage of the disulfide bridges (Figure 1d). This success highlights the feasibility of the designed controllable conjugation and deconjugation protocol.

In this study, we describe a unique method for controllable protein conjugation and deconjugation. The conjugates can be synthesized easily from commercially available materials, and the reversible tactic constructs bicyclic structures on the peptide and protein by a simple, easy to obtain motif. The conjugation protocol allows for attachment to the primary amine of the polypeptide under physiological conditions, and the deconjugation procedure remains mild and specific. The conjugation demonstrates reliable stability on several different peptide systems exhibiting wide side-chain tolerance. Finally, we have illustrated the ability to fine-tune deconjugation conditions to achieve complete restoration of specific polypeptide sequences, offering a practical and effective method for sophisticated protein modification.

## Data Availability

The data underlying this study are available in the published article and its Supporting Information.

## Supporting Information Available

Experimental details including the synthetic procedures and analytical data for all new compounds, copies of HPLC traces, and NMR spectra of the compounds (PDF)
