# Supplementary material for: Reversible Conjugation of Polypeptides and Proteins Utilizing a [3.3.1] Scaffold under Mild Conditions
Source: Org Lett. 2024 Jul 22;26(30):6428–32. doi: 10.1021/acs.orglett.4c02228 (PMC11301669; doi:10.1021/acs.orglett.4c02228)
Supplement: Supplementary file 1 — ol4c02228_si_001.pdf [file ol4c02228_si_001.pdf]

Supporting Information

Reversible Conjugation of Polypeptides and Proteins Utilizing a [3.3.1] Scaffold under Mild Conditions.

Ryan J. Bartlett, Kelly D. Crisostomo, and Qiang Zhang*
Department of Chemistry, University at Albany, State University of New York
1400 Washington Avenue, Albany, NY 12222 (USA)
E-mail: qzhang5@albany.edu

Table of Contents:

I. General Information. **Page 2**
II. General Procedure. **Page 3**
III. Synthesis of Mercaptan Conjugates. **Page 3**
IV. Screening of Bicylic Formation on 12. **Page 6**
V. Investigation of 6 Conjugation Scope. **Page 10**
VI. Investigation of Deconjugation Scope. **Page 20**
VII. Conjugation to Recombinant Insulin. **Page 28**
VIII. Deconjugation from Insulin. **Page 32**
IX. NMR Spectra. **Page 34**
X. References. **Page 36**

I. General Information.

Reagents were purchased from Sigma-Aldrich, Ambeed, and Matrix Scientific. Purchased compounds were utilized without purification. Reactions involving small molecule synthesis were performed in oven-dried glassware equipped with a medium Teflon stir bar sealed under argon unless stated otherwise. Analytical TLC was performed on Supelco silica gel 60 F254 plates and flash column chromatography was performed on SiliCycle SiliaFlash® 60 (40–63 µm). Reactions involving peptides and proteins were carried out in Fisherbrand (12 x 35) mm, 0.5 Dr. threaded vials capped tightly equipped with a small Teflon stir bar unless stated otherwise. Reactions were heated by a sand bath attached to a Powerstat® variac and temperatures reported correspond to monitored heat of the sand bath by glass thermometer. pH values were monitored and reported using Whatman™ Type CS pH Indicator Papers for ranges pH 6.0-8.1 and pH 5.2-6.8 in tandem. Recombinant Insulin was purchased from MP Biomedicals and used without purification. Unless noted, all yields reported refer to chromatographically pure materials.

^{1}H NMR spectra were recorded at 500 MHz at ambient temperature with dimethyl sulfoxide-d6 (DMSO-d6) (Cambridge Isotope Laboratories, Inc., and Oakwood Chemical) and Chloroform-d ($CDCl_3$-d) (Cambridge Isotope Laboratories, Inc., and Oakwood Chemical) solvents. Chemical shifts are reported in parts per million relatives to DMSO-d6 (1H, δ 2.50) and $CDCl_3$-d (1H, δ 7.26). ^{13}C NMR spectra were recorded at 125 MHz at ambient temperature with dimethyl sulfoxide-d6 (DMSO-d6) (Cambridge Isotope Laboratories, Inc., and Oakwood Chemical) and Chloroform-d ($CDCl_3$-d) (Cambridge Isotope Laboratories, Inc., and Oakwood Chemical) solvents. Chemical shifts are reported in parts per million relatives to DMSO-d6 (13C, δ 35.51) and $CDCl_3$-d (13C, δ 77.36).

Liquid Chromatography-Mass Spectroscopy (LC-MS) via electrospray ionization (ESI) mode were obtained using an Agilent G6530BA Q-TOF Mass Spectrometer equipped with a Higgins Analytical CLIPEUS 5 µm 250 ×4.6 mm C18 column. Flow rate followed 0.6 mL/min with a gradient of 10 %-60 % MeCN, 1 % Formic acid (against H_2O, 1 % Formic acid) for 20 minutes.

1,4-Dithiane-2,5-diol (CAS [40018-26-6]) (**10**) and 2,5-Dihydroxy-2,5-dimethyl-1,4-dithiane (CAS [55704-78-4]) (**11**) were purchased from commercial sources. Synthesis of the benzaldehyde dimer (**3**) began with commercially available 2-Mercaptobenzoic acid (CAS [147-93-3]) (**1**). Synthesis of heterocyclic dimers (**6** and **9**) began with commercially available 2-Mercaptonicotinic acid (CAS [38521-46-9]) (**4**) and Methyl 5-bromo-2-chloronicotinate (CAS [78686-79-0]) (**7**).

II. General Procedure.

Automated solid phase peptide synthesis was performed using Biotage Initiator+ Alstra automated microwave peptide synthesizer. Fmoc-protected amino acids were deblocked using DBU/Piperidine/DMF (2/2/96 *v/v/v*) at room temperature for 7 minutes. Coupling was performed with HATU (4.5 eq), DIPEA (9 eq), and sequence amino acid (AA) (4.2 eq) at 40 °C for 9 minutes. Synthesized peptides underwent global deprotection and cleavage from the resin using a TFA/H_2O/TIPS (95/2.5/2.5 *v/v/v*) cocktail that stirred at room temperature for 2-4 hours. Excess TFA was then blown away via argon stream.

Synthesized peptides were purified by Dionex Ultimate UHPLC system equipped with a UV detector and a Higgins Analytical CLIPEUS 5 µm 250×10 mm C18 column. Flow rate followed 3.5 mL/min with a gradient of 10 %-70 % MeCN, 0.5 % TFA (solvent B) (against H_2O, 0.5 % TFA (solvent A)).

Purification of conjugated peptides ill-suited for HPLC was performed using Biotage Isolute C2(EC) in a flash-suitable column pushed through by house air pressure and double-distilled H_2O and MeCN mobile phase without acid additive. Column measured 10 cm length and eluent was collected in twelve 6 mL fractions with increasing ration of MeCN (0 %, 10 %-25 %, increasing by 5 % for four fractions. 30 %-90 %, increasing by 10 % for seven fractions). Powder was obtained by lyophilization in a Labconco FreeZone 1 Benchtop Freeze Dryer Lyophilizer after being frozen in solution by liquid N_2.

III. Synthesis of Mercaptan Conjugates.

Synthesis of ***3***

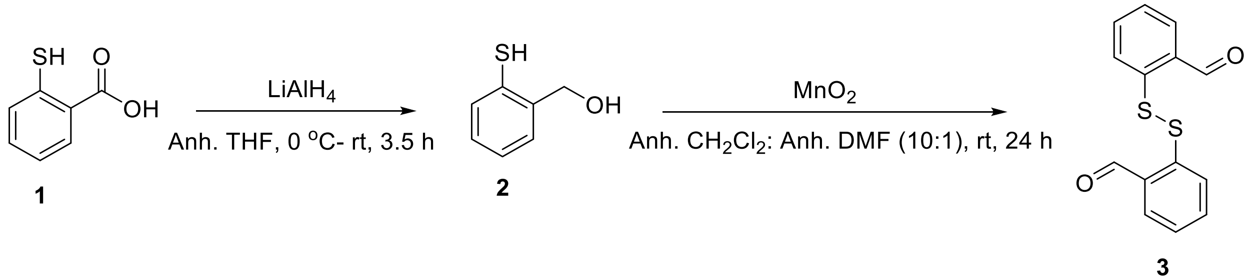

$LiAlH_4$ (246 mg, 6.48 mmol) was stirred into a slurry within a flame dried flask with anhydrous THF (5 mL) under argon. The solution was cooled to 0 °C in an ice bath before mercapto-benzoic acid **1** (250 mg, 1.62 mmol, 0.32 M) dissolved in anhydrous THF was added dropwise via syringe. The reaction was warmed to room temperature and stirred under argon for 3.5 hours, monitored by TLC. The completed reaction was cooled to 0 °C and quenched by 2 mL of saturated NH_4Cl added dropwise to the solution. The solution was filtrated through Celite® 545 and subsequently washed by ethyl acetate. The combined ethyl acetate

fractions were concentrated via vacuo and afforded white solid (60 mg, 0.428 mmol), which was used directly in the next step without purification.

A flask containing MnO_2 (369 mg, 4.28 mmol) and stir bar was activated overnight in a 120 °C oven. The flask was removed from the oven and placed under vacuum to cool. Under argon atmosphere, the MnO_2 was mixed with anhydrous CH_2Cl_2 (5 mL). In parallel, the crude alcohol **2** (60 mg, 0.428 mmol, 0.09 M) was dissolved in 0.5 mL of anhydrous DMF and was added to the MnO_2 solution by syringe. The reaction mixture was stirred for 24 hours at room temperature, and the completed reaction was passed through Celite® 545 followed by CH_2Cl_2 wash. The combined CH_2Cl_2 fractions were concentrated via vacuo and the oily residue was purified by silica gel column (CH_2Cl_2), affording 56 mg (0.204 mmol) of **3** as a white powder (25 % yield via two step). R_f= 0.82 (CH_2Cl_2: MeOH, 30:1)

^{1}H NMR (500 MHz, $CDCl_3$-d) δ 10.22 (s, 2H), 7.87 (dd, J = 7.5, 0.8 Hz, 2H), 7.78 (d, J = 8.0 Hz, 2H), 7.49 (td, J = 8.2, 1.0 Hz, 2H), 7.39 (t, J = 7.4 Hz, 2H). Analysis results correspond with previously reported data.[1]

*Synthesis of **6***

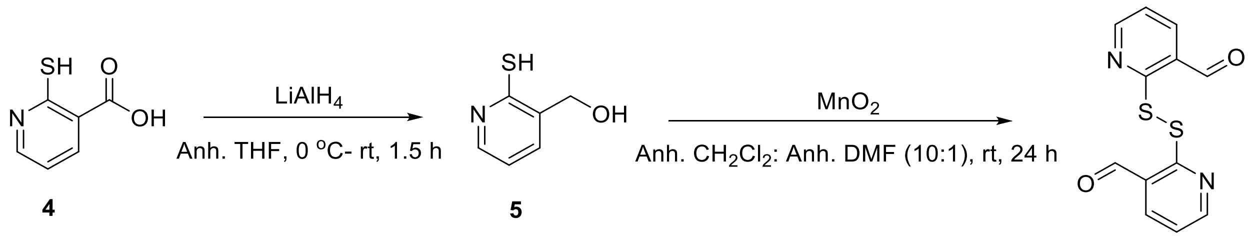

$LiAlH_4$ (319 mg, 8.40 mmol) was stirred into a slurry within a flame dried flask with anhydrous THF (12 mL) under argon. The solution was cooled to 0 °C in an ice bath before mercapto-nicotinic acid **4** (466 mg, 3.00 mmol, 0.25 M) dissolved in anhydrous THF was added dropwise via syringe. The reaction was warmed to room temperature and stirred under argon for 1.5 hours, monitored by TLC. The completed reaction was cooled to 0 °C and quenched by 3 mL of saturated NH_4Cl added dropwise to the solution. The solution was filtrated through Celite® 545 and subsequently washed by 10 % MeOH in CH_2Cl_2. The combined MeOH: CH_2Cl_2 fractions were concentrated via vacuo and afforded orange solid (265 mg, 1.88 mmol), which was used directly in the next step without purification.

A flask containing MnO_2 (4.1 g, 46.92 mmol) and stir bar was activated overnight in a 120 °C oven. The flask was removed from the oven and placed under vacuum to cool. Under argon atmosphere, the MnO_2

was mixed with anhydrous CH_2Cl_2 (22 mL). In parallel, the crude alcohol **5** (265 mg, 1.88 mmol, 0.09M) was dissolved in 2 mL of anhydrous DMF and was added to the MnO_2 solution by syringe. The reaction mixture was stirred for 24 hours at room temperature, and the completed reaction was passed through Celite® 545 followed by 10 % MeOH in CH_2Cl_2 wash. The combined MeOH: CH_2Cl_2 fractions were concentrated via vacuo and the oily residue was purified by silica gel column (Hexane: Ethyl Acetate 1:1), affording 113 mg (0.408 mmol) of **6** as a yellow powder (27 % yield via two step). R_f= 0.26 (Hexane: Ethyl Acetate, 1:1)

^{1}H NMR (500 MHz, $CDCl_3$-d) δ 10.39 (s, 2H), 8.56 (dd, *J* = 4.6, 1.4 Hz, 2H), 8.09 (dd, *J* = 7.5, 1.4 Hz, 2H), 7.27 (dd, *J* = 7.5, 4.8 Hz, 2H). **^{13}C NMR** (125 MHz, $CDCl_3$-d) δ 190.8, 160.2, 153.8, 140.4, 130.3, 121.4. **IR** (FTIR) cm^{-1} 2923, 2853, 1677, 1576, 1546, 1444, 1412, 1378, 1258, 1239, 1202, 1126, 1080, 1065, 844, 788, 738, 730, 682, 651. **HRMS** (ESI-QTOF) m/z: $[M+H]^+$ Calcd for $C_{12}H_9N_2O_2S_2$ 277.0099; Found 277.0096

*Synthesis of **8***

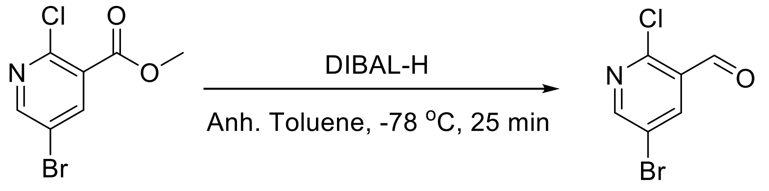

7 **8**

To a flame-dried flask with stir bar was bromo-chloronicotinate **7** (500 mg, 2.00 mmol, 0.27 M) dissolved in anhydrous toluene (7.5 mL) added. Under argon, the solution was cooled to -78 °C using an acetone/dry ice bath. DIBAL-H (3.00 mmol, 1.2 M in toluene (2.5 mL)) was added dropwise to the solution via syringe. The solution was stirred for 25 minutes at -78 °C, monitored by TLC. The reaction was quenched with H_2O, 15% NaOH, and H_2O again, (*N, N, 3N*). The product aldehyde **8** was purified by silica gel column (Hexane: Ethyl Acetate 9:1) and concentrated to afford 208 mg (0.944 mmol) as a white dust (47 % yield). R_f= 0.54 (Hexane: Ethyl Acetate, 6:1)

^{1}H NMR (500 MHz, $CDCl_3$-d) δ 10.37 (s, 1H), 8.66 (d, *J* = 2.5 Hz, 1H), 8.32 (d, *J* = 2.6 Hz, 1H). Analysis matches the commercially available compound spectra.

*Synthesis of **9***

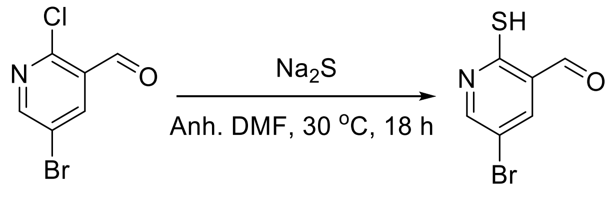

To a small flask was **8** (42 mg, 0.19 mmol, 0.13 M) and Na_2S (30 mg, 0.38 mmol) added. The flask was placed under vacuum and exchanged with argon before anhydrous DMF (1.5 mL) was added via syringe at room temperature. The flask was stirred in a sand bath at 30 °C for 18 hours, monitored by TLC. The reaction solution was brought down to ~pH 3 with 1M HCl and was extracted with ethyl acetate. The organic phase was washed with H_2O and dried by Na_2SO_4. The solution was concentrated via vacuo and the oily residue was purified by silica gel column (Hexane: Ethyl Acetate, 1:1) to afford 9 mg (0.04 mmol) of **9** as an orange powder (21 % yield). R_f= 0.13 (Hexane: Ethyl Acetate, 1:1)

^{1}H NMR (500 MHz, DMSO d-6) δ 10.44 (s, 1H), 8.24 (d, *J* = 2.4 Hz, 1H), 7.84 (d, *J* = 2.4 Hz, 1H). **^{13}C NMR** (125 MHz, DMSO d-6) δ 186.5, 174.2, 140.5, 136.4, 131.2, 101.2. **IR** (FTIR) cm^{-1} 3057, 2906, 2823, 2770, 1674, 1573, 1303, 1235, 1147. **HRMS** (ESI-QTOF) m/z: $[M+H]^+$ Calcd for C_6H_5BrNOS 217.9269; Found 217.9252

IV. Screening of Bicylic Formation on 12.

*Conjugation of **12** to **13***

Chemical Formula: $C_{77}H_{111}N_{21}O_{24}S_2$
Exact Mass: 1777.75523
Molecular Weight: 1778.97800

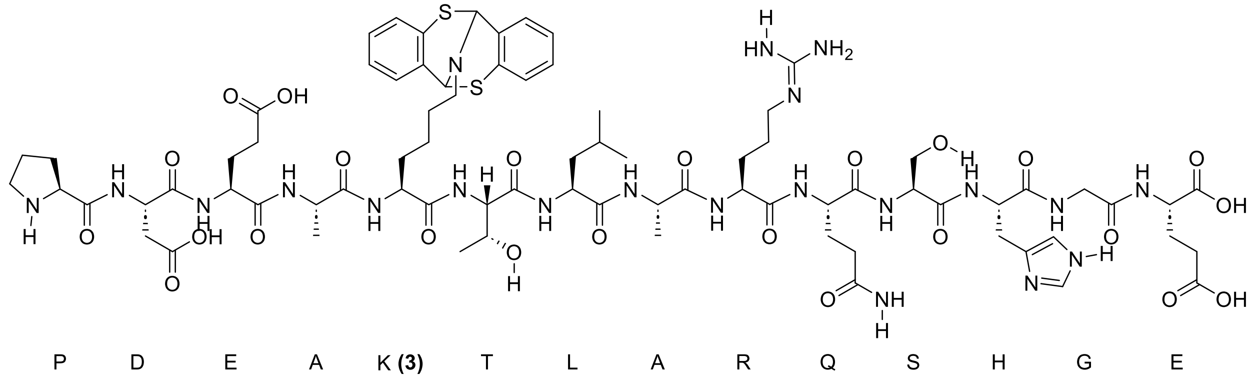

13

Peptide **12** was prepared by automated SPPS. 4.1 mg (0.0148 mmol) of **3** was dissolved in 104 µL of 0.5 M TCEP•HCl solution (0.0518 mmol) in H_2O for 20 minutes at 38 °C. 5.7 mg (0.0037 mmol, 12 mM) of **12** was dissolved in 200 µL of PBS buffer and administered directly into the solution. The reaction was adjusted to a pH of 6.3 and was stirred at 38 °C until bicyclic-formation was observed 24 hours later. The conjugated peptide was purified by preparative HPLC (10 to 70% solvent B over 20 min, Higgins Analytical CLIPEUS 5 µm 250×10 mm C18 column) and lyophilized to afford 3.5 mg (0.0020 mmol) of **13** as a fluffy, white solid (53% yield). HPLC (Higgins Analytical CLIPEUS 5 µm 250×4.6 mm C18 column, water/acetonitrile = 90/10 to 40/60 over 20 min, flow rate = 0.600 mL/min, l = 214 nm) tR = 11.94 min. HRMS (ESI-QTOF) m/z: $[M+3H]^{3+}$ Calcd for $C_{77}H_{114}N_{21}O_{24}S_2$ 593.5924; Found 593.5934

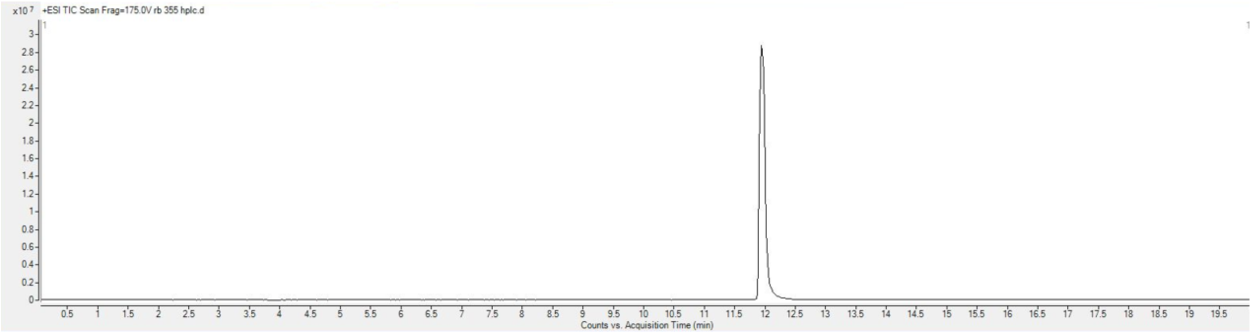

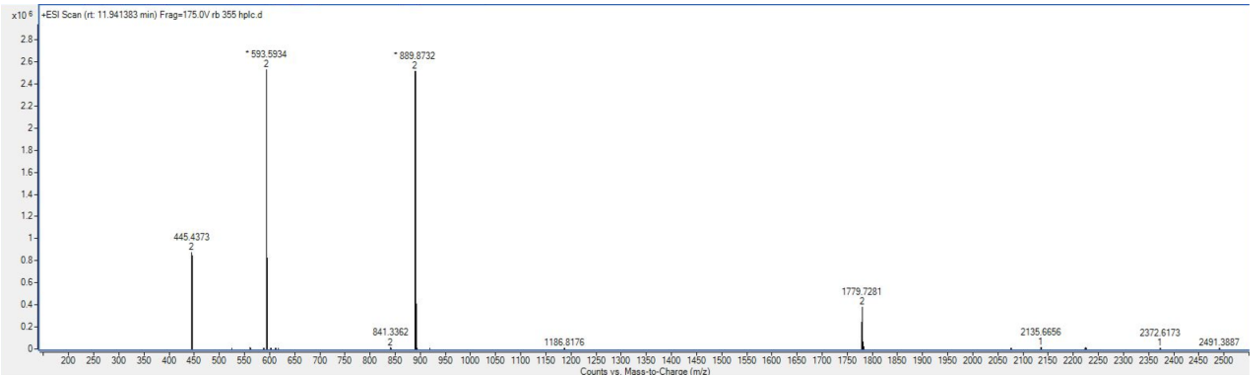

*Conjugation of **12** to **14***

Chemical Formula: $C_{75}H_{109}N_{23}O_{24}S_2$
Exact Mass: 1779.74572
Molecular Weight: 1780.95400

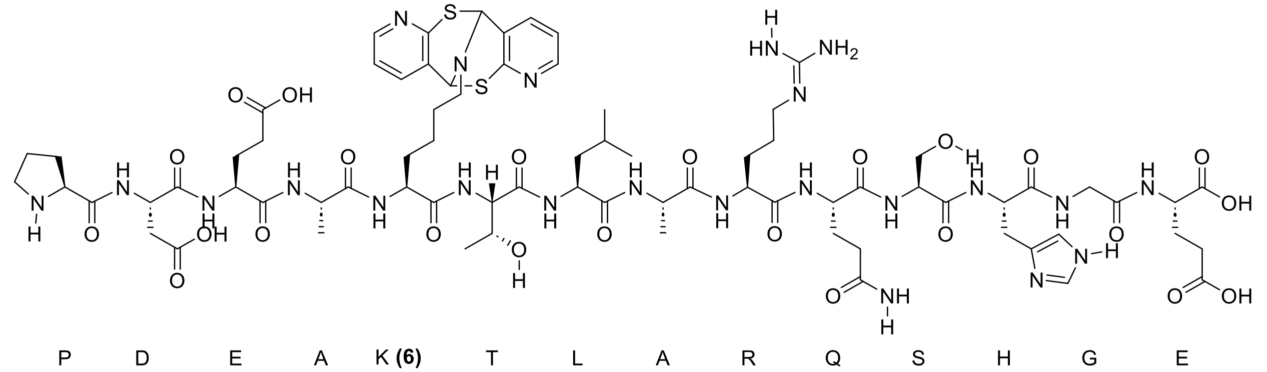

14

4.1 mg (0.0148 mmol) of **6** was dissolved in 104 µL of 0.5 M TCEP•HCl solution (0.0518 mmol) in H_2O for 20 minutes at 38 °C. 5.7 mg (0.0037 mmol, 12 mM) of **12** was dissolved in 200 µL of PBS buffer and administered directly into the solution. The reaction was adjusted to a pH of 6.3 and was stirred at 38 °C until bicyclic-formation was observed 48 hours later. Due to instability during preparative HPLC, the product was isolated using C2 reverse-phase flash column procedure described in **II. General Procedure**. Collected in twelve 6 mL fractions, isolated product was lyophilized to afford 6.0 mg (0.0034 mmol) of **14** as a fluffy, off-white solid (91% yield). HPLC (Higgins Analytical CLIPEUS 5 µm 250×4.6 mm C18 column, water/acetonitrile = 90/10 to 40/60 over 20 min, flow rate = 0.600 mL/min, l = 214 nm) tR = 8.32 min. HRMS (ESI-QTOF) m/z: $[M+2H]^{2+}$ Calcd for $C_{75}H_{111}N_{23}O_{24}S_2$ 890.8801; Found 890.8817

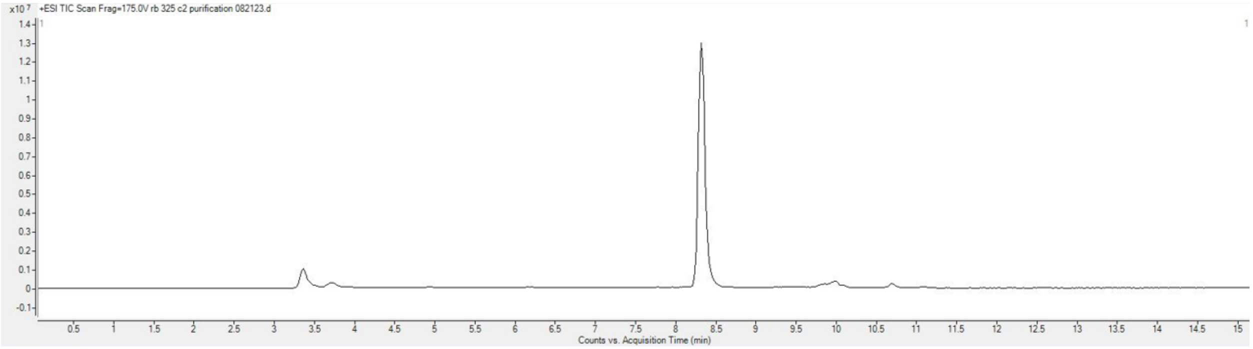

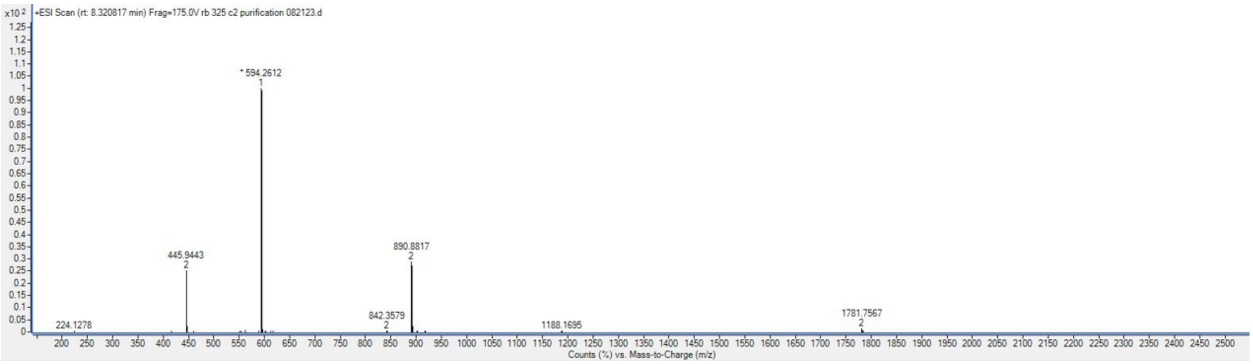

*Conjugation of **12** to **15***

Chemical Formula: $C_{67}H_{107}N_{21}O_{24}S_2$
Exact Mass: 1653.72393
Molecular Weight: 1654.83600

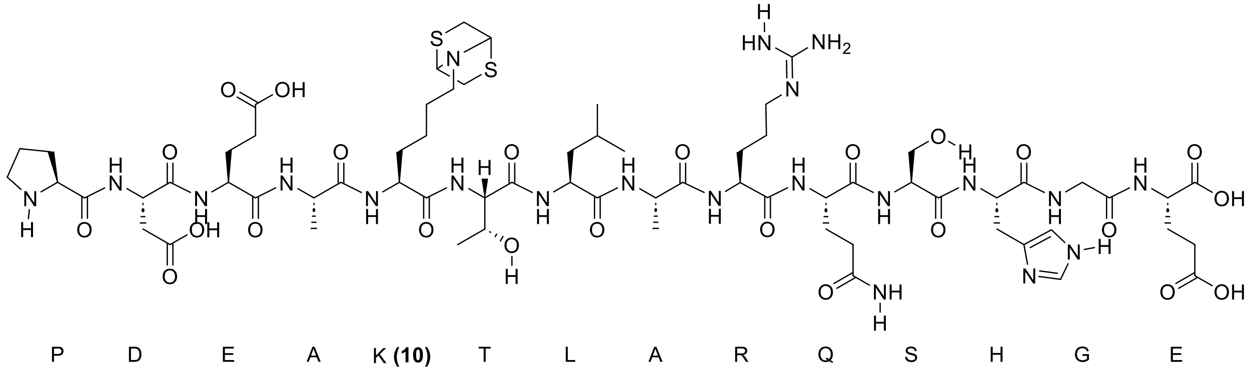

15

2.3 mg (0.0148 mmol) of **10** and 5.7 mg (0.0037 mmol, 12 mM) of **12** were dissolved in 300 µL of PBS buffer. The reaction was adjusted to a pH of 6.3 and was stirred at 38 °C until bicyclic-formation was observed 24 hours later. The conjugated peptide was purified by preparative HPLC (10 to 70% solvent B over 20 min, Higgins Analytical CLIPEUS 5 µm 250×10 mm C18 column) and lyophilized to afford 2.0 mg (0.0012 mmol) of **15** as a fluffy, white solid (33 % yield). HPLC (Higgins Analytical CLIPEUS 5 µm

250×4.6 mm C18 column, water/acetonitrile = 90/10 to 40/60 over 20 min, flow rate = 0.600 mL/min, l = 214 nm) tR = 7.73 min. HRMS (ESI-QTOF) m/z: $[M+3H]^{3+}$ Calcd for $C_{67}H_{110}N_{21}O_{24}S_2$ 552.2486; Found 552.2407

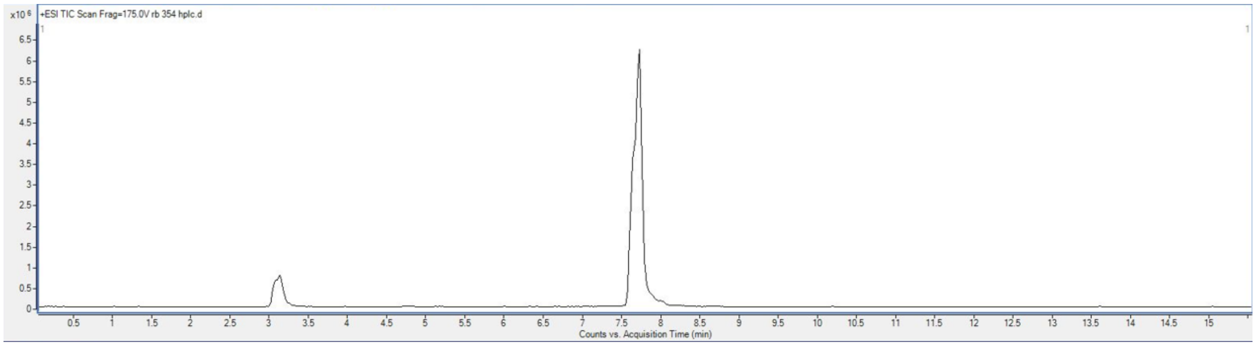

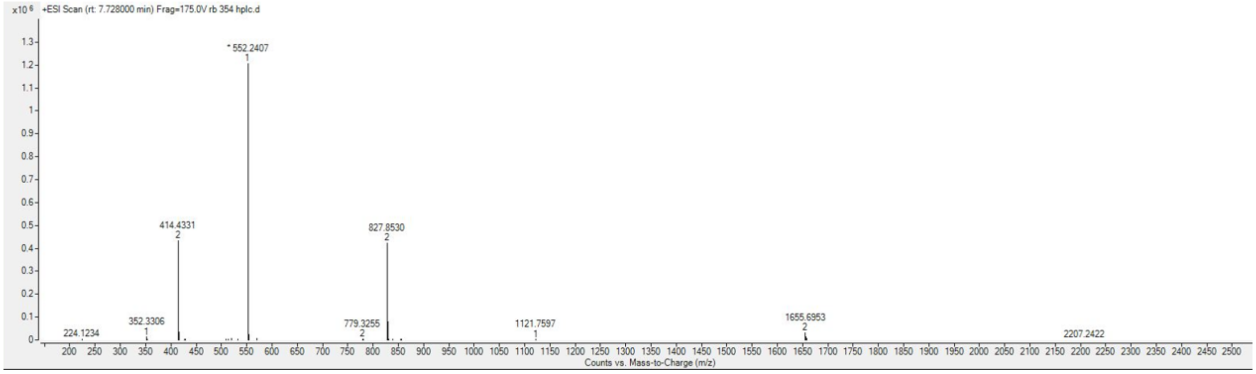

V. Investigation of 6 Conjugation Scope.

*Conjugation of **18** to **27***

Chemical Formula: $C_{74}H_{112}N_{22}O_{20}S_2$
Exact Mass: 1692.78647
Molecular Weight: 1693.96400

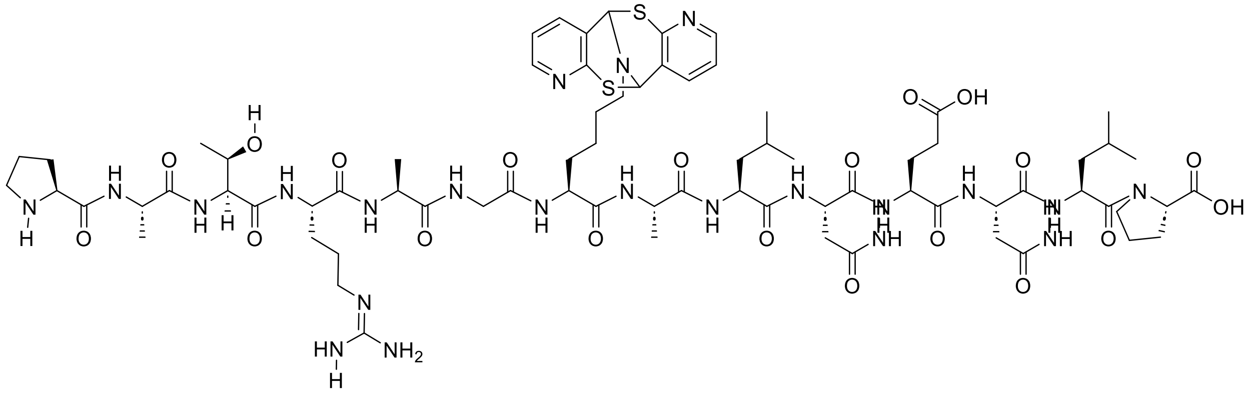

P A T R A G K **(6)** A L N E N L P

27

Peptide **18** was prepared by automated SPPS. 4.1 mg (0.0148 mmol) of **6** was dissolved in 104 µL of 0.5 M TCEP•HCl solution (0.0518 mmol) and was stirred at 38 °C for 20 minutes. 5.4 mg (0.0037 mmol, 12 mM) of **18** was dissolved in 200 µL of PBS buffer and administered directly into the solution. The reaction was adjusted to a pH of 6.3 and was stirred at 38 °C until bicyclic-formation was observed 24 hours later. Due to instability during preparative HPLC, the product was isolated using C2 reverse-phase flash column procedure described in **II. General Procedure**. Collected in twelve 6 mL fractions, isolated product was lyophilized to afford 2.1 mg (0.0012 mmol) of **27** as a fluffy, white solid (34 % yield). HPLC (Higgins Analytical CLIPEUS 5 µm 250×4.6 mm C18 column, water/acetonitrile = 90/10 to 40/60 over 20 min, flow rate = 0.600 mL/min, l = 214 nm) tR = 10.83 min. HRMS (ESI-QTOF) m/z: $[M+3H]^{3+}$ Calcd for $C_{74}H_{115}N_{22}O_{20}S_2$ 565.2694; Found 565.2632

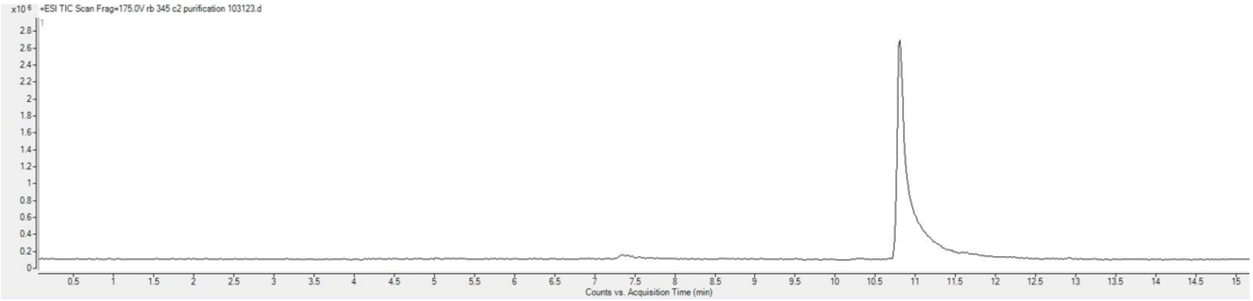

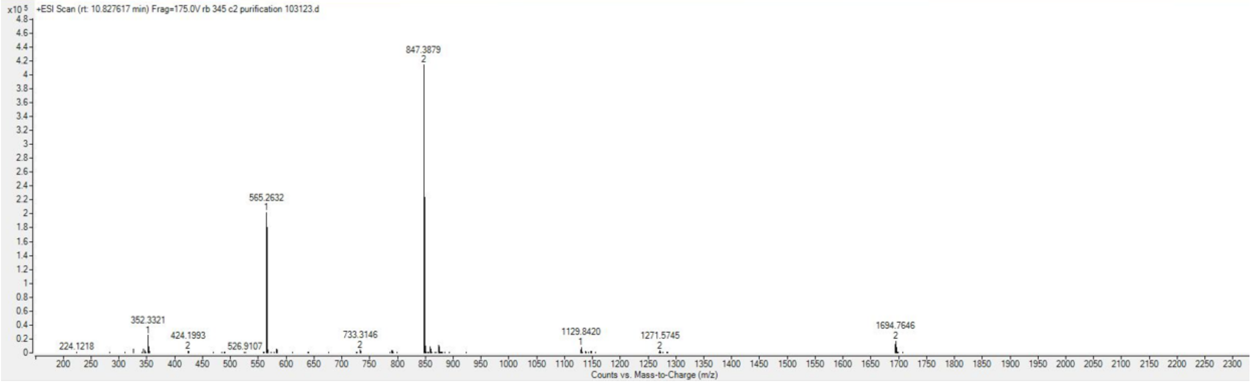

*Conjugation of **20** to **29***

Chemical Formula: $C_{57}H_{84}N_{14}O_{12}S_3$
Exact Mass: 1252.55553
Molecular Weight: 1253.56500

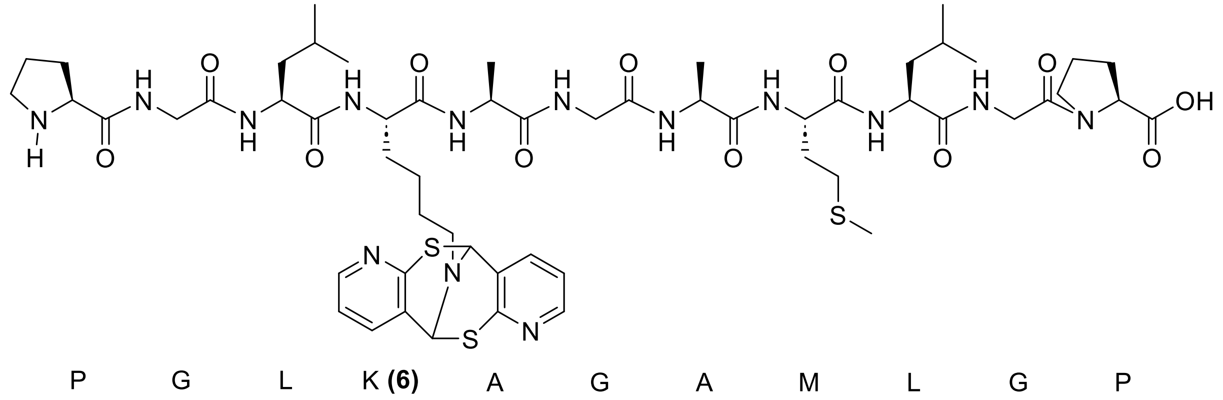

29

Peptide **20** was prepared by automated SPPS. 2.5 mg (0.0089 mmol) of **6** was dissolved in 104 µL of 0.5 M TCEP•HCl solution (0.0518 mmol) and was stirred at 38 °C for 20 minutes. 3.0 mg (0.003 mmol, 10 mM) of **20** was dissolved in 200 µL of PBS buffer and administered directly into the solution. The reaction was adjusted to a pH of 6.3 and was stirred at 38 °C until bicyclic-formation was observed 38 hours later. Due to instability during preparative HPLC, the product was isolated using C2 reverse-phase flash column procedure described in **II. General Procedure**. Collected in twelve 6 mL fractions, isolated product was lyophilized to afford 3.2 mg (0.0026 mmol) of **29** as a light, white powder (84 % yield). HPLC (Higgins Analytical CLIPEUS 5 µm 250×4.6 mm C18 column, water/acetonitrile = 90/10 to 40/60 over 20 min, flow rate = 0.600 mL/min, l = 214 nm) tR = 10.50 min. HRMS (ESI-QTOF) m/z: $[M+2H]^{2+}$ Calcd for $C_{57}H_{86}N_{14}O_{12}S_3$ 627.2850; Found 627.2844

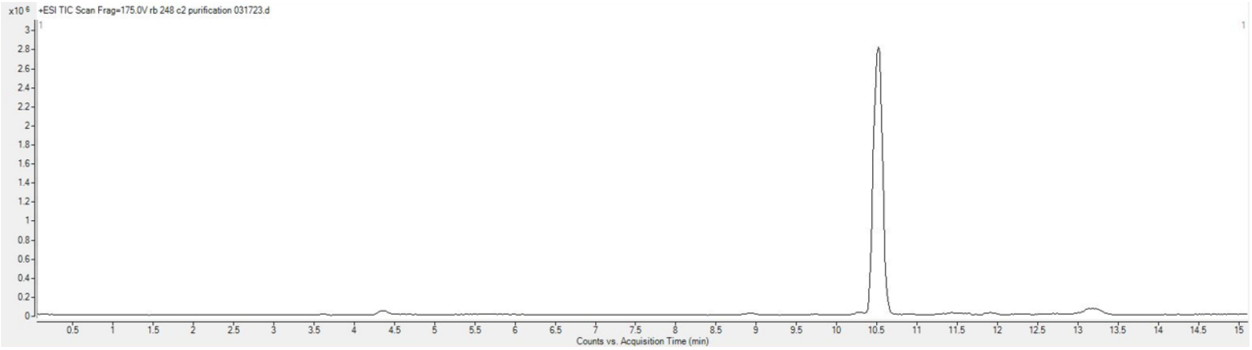

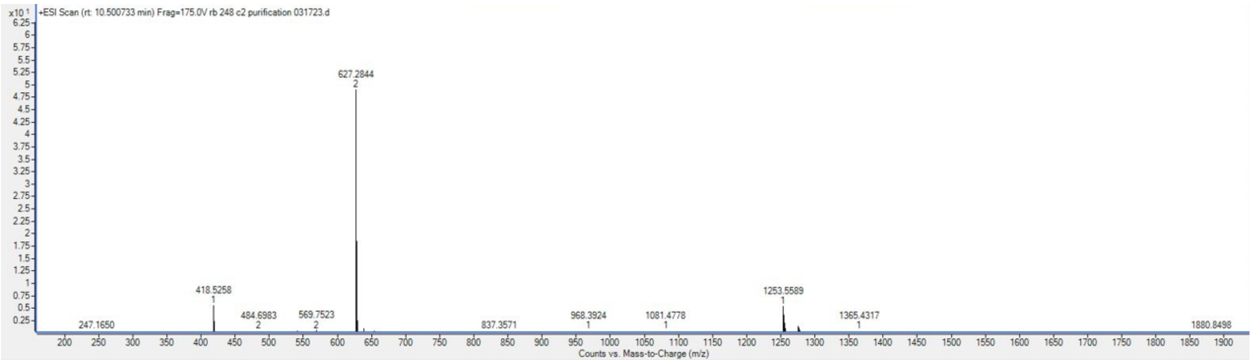

*Conjugation of **21** to **30***

Chemical Formula: $C_{65}H_{102}N_{18}O_{16}S_2$
Exact Mass: 1454.71626
Molecular Weight: 1455.76100

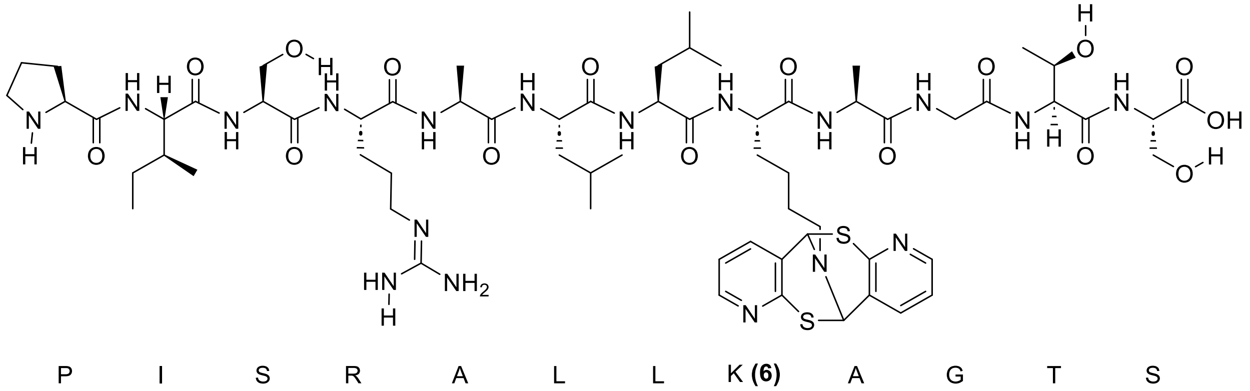

30

Peptide **21** was prepared by automated SPPS. 4.1 mg (0.0148 mmol) of **6** was dissolved in 104 µL of 0.5 M TCEP•HCl solution (0.0518 mmol) and was stirred at 38 °C for 20 minutes. 4.5 mg (0.0037 mmol, 12 mM) of **21** was dissolved in 200 µL of PBS buffer and administered directly into the solution. The reaction was adjusted to a pH of 6.3 and was stirred at 38 °C until bicyclic-formation was observed 24 hours later. Due to instability during preparative HPLC, the product was isolated using C2 reverse-phase flash column procedure described in **II. General Procedure**. Collected in twelve 6 mL fractions, isolated product was lyophilized to afford 5.0 mg (0.0034 mmol) of **30** as a flakey, white solid (93% yield). HPLC (Higgins Analytical CLIPEUS 5 µm 250×4.6 mm C18 column, water/acetonitrile = 90/10 to 40/60 over 20 min, flow rate = 0.600 mL/min, l = 214 nm) tR = 11.53 min. HRMS (ESI-QTOF) m/z: $[M+3H]^{3+}$ Calcd for $C_{65}H_{105}N_{18}O_{16}S_2$ 485.9127; Found 485.9075

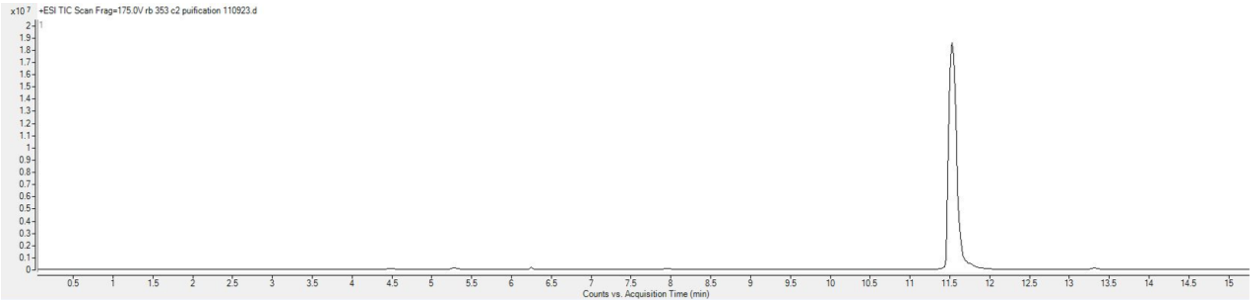

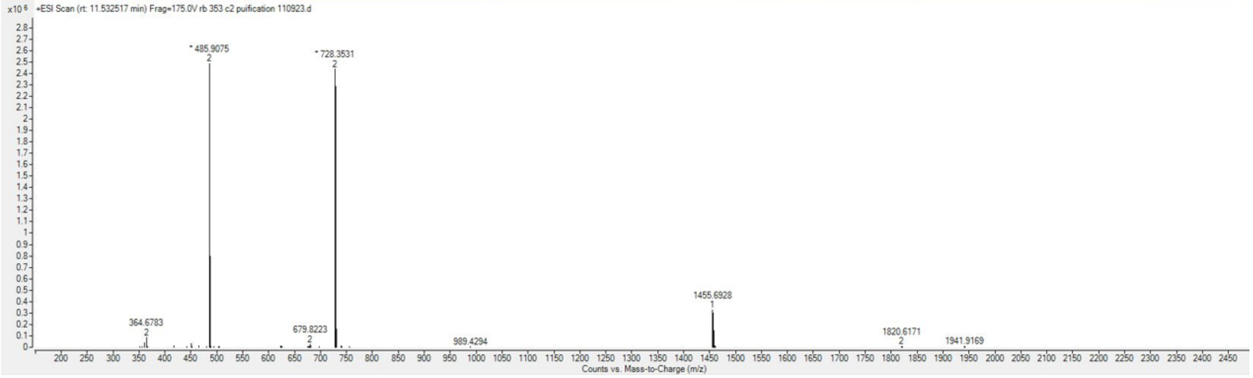

*Conjugation of **22** to **31***

Chemical Formula: $C_{63}H_{85}N_{17}O_{16}S_2$
Exact Mass: 1399.58016
Molecular Weight: 1400.59600

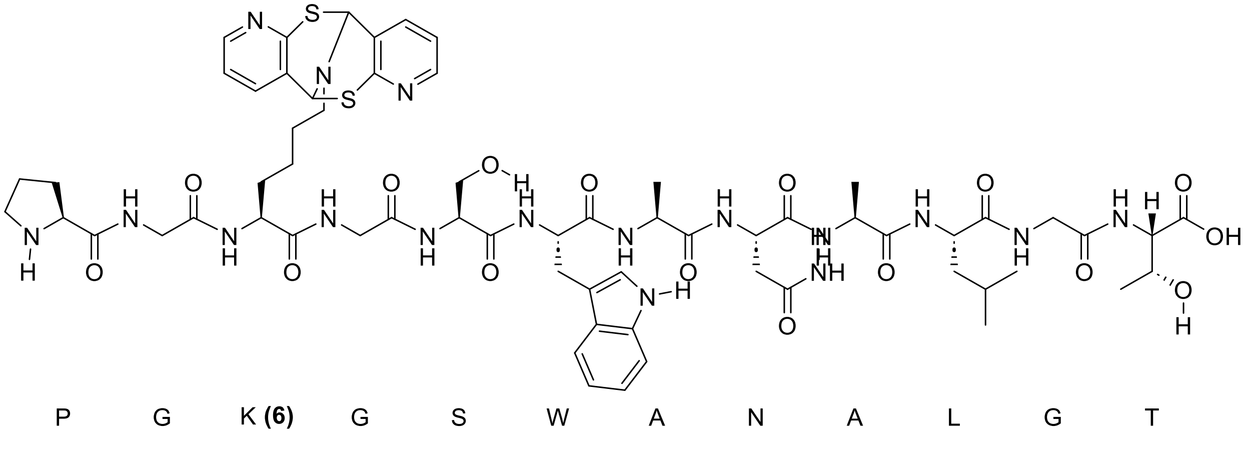

31

Peptide **22** was prepared by automated SPPS. 4.1 mg (0.0148 mmol) of **6** was dissolved in 104 µL of 0.5 M TCEP•HCl solution (0.0518 mmol) and was stirred at 38 °C for 10 minutes. 4.0 mg (0.0035 mmol, 12

mM) of **22** was dissolved in 200 µL of PBS buffer and administered directly into the solution. The reaction was adjusted to a pH of 6.3 and was stirred at 38 °C until bicyclic-formation was observed 18 hours later. Due to instability during preparative HPLC, the product was isolated using C2 reverse-phase flash column procedure described in **II. General Procedure**. Collected in twelve 6 mL fractions, isolated product was lyophilized to afford 2.7 mg (0.0019 mmol) of **31** as a fluffy, white solid (55%). HPLC (Higgins Analytical CLIPEUS 5 µm 250×4.6 mm C18 column, water/acetonitrile = 90/10 to 40/60 over 20 min, flow rate = 0.600 mL/min, l = 214 nm) tR = 9.82 min. HRMS (ESI-QTOF) m/z: $[M+H]^+$ Calcd for $C_{63}H_{86}N_{17}O_{16}S_2$ 1400.5874; Found 1400.5869

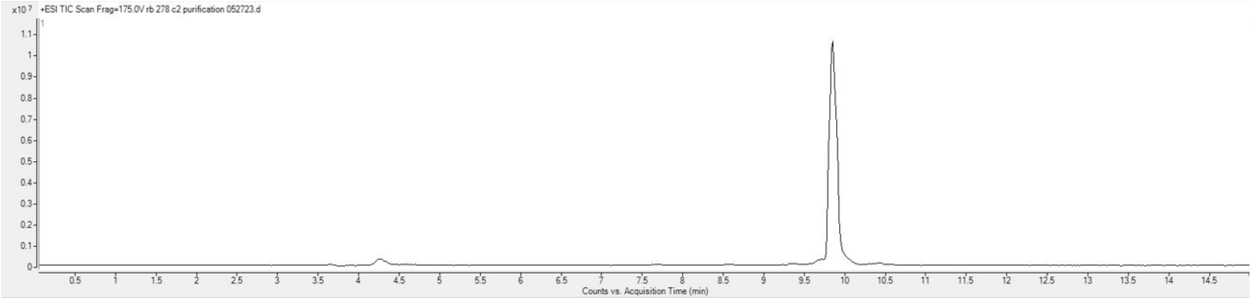

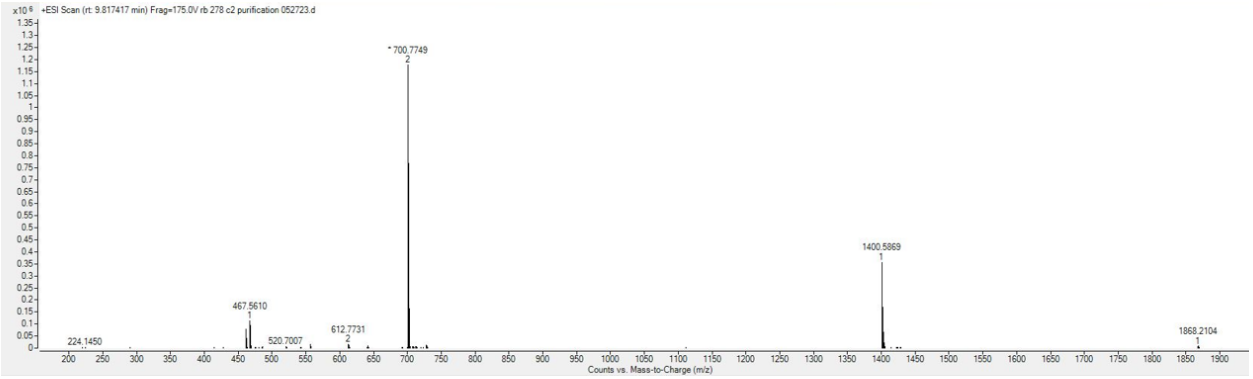

*Conjugation of **23** to **32***

Chemical Formula: $C_{84}H_{124}N_{26}O_{24}S_3$
Exact Mass: 1976.84439
Molecular Weight: 1978.25400

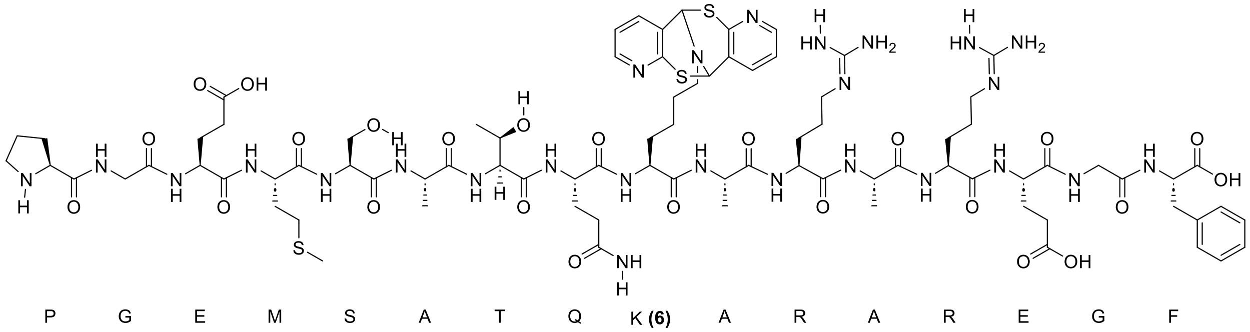

32

Peptide **23** was prepared by automated SPPS. 4.1 mg (0.0148 mmol) of **6** was dissolved in 104 µL of 0.5 M TCEP•HCl solution (0.0518 mmol) and was stirred at 38 °C for 20 minutes. 6.4 mg (0.0037 mmol, 12 mM) of **23** was dissolved in 200 µL of PBS buffer and administered directly into the solution. The reaction was adjusted to a pH of 6.3 and was stirred at 38 °C until bicyclic-formation was observed 21 hours later. Due to instability during preparative HPLC, the product was isolated using C2 reverse-phase flash column procedure described in **II. General Procedure**. Collected in twelve 6 mL fractions, isolated product was lyophilized to afford 1.2 mg (0.0006 mmol) of **32** as a crumbly, white solid (16% yield). HPLC (Higgins Analytical CLIPEUS 5 µm 250×4.6 mm C18 column, water/acetonitrile = 90/10 to 40/60 over 20 min, flow rate = 0.600 mL/min, l = 214 nm) tR = 8.74 min. HRMS (ESI-QTOF) m/z: $[M+3H]^{3+}$ Calcd for $C_{84}H_{127}N_{26}O_{24}S_3$ 659.9554; Found 659.9568

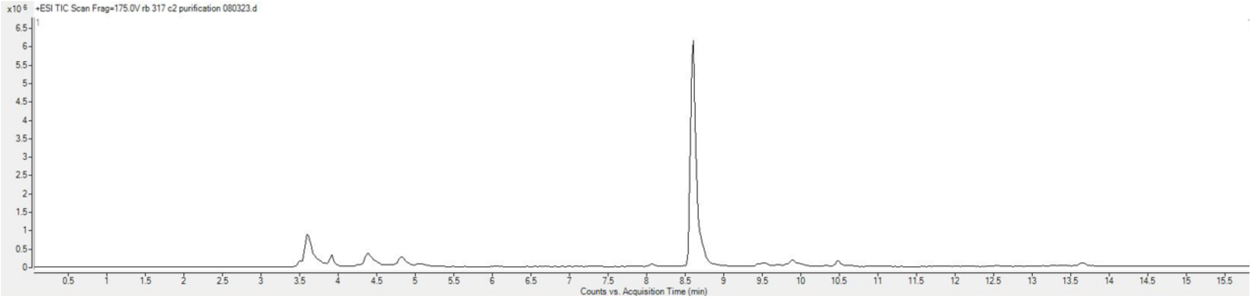

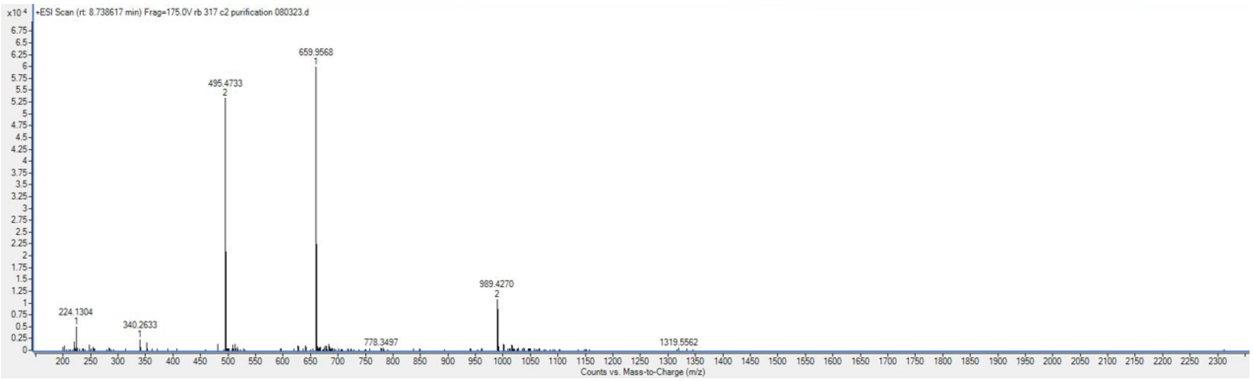

*Conjugation of **25** to **34***

Chemical Formula: $C_{60}H_{88}N_{16}O_{14}S_2$
Exact Mass: 1320.61073
Molecular Weight: 1321.58200

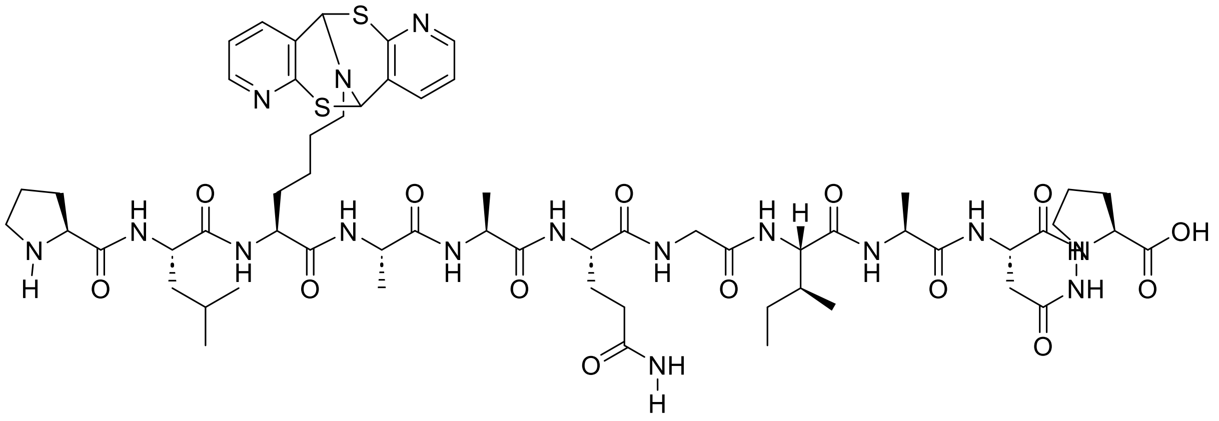

P L K **(6)** A A Q G I A N P

34

Peptide **25** was prepared by automated SPPS. 4.1 mg (0.0148 mmol) of **6** was dissolved in 104 µL of 0.5 M TCEP•HCl solution (0.0518 mmol) and was stirred at 38 °C for 10 minutes. 4.0 mg (0.0037 mmol, 12 mM) of **25** was dissolved in 200 µL of PBS buffer and administered directly into the solution. The reaction was adjusted to a pH of 6.3 and was stirred at 38 °C until bicyclic-formation was observed 18 hours later. Due to instability during preparative HPLC, the product was isolated using C2 reverse-phase flash column procedure described in **II. General Procedure**. Collected in twelve 6 mL fractions, isolated product was lyophilized to afford 3.4 mg (0.0025 mmol) of **34** as a fluffy, white solid (68% yield). HPLC (Higgins Analytical CLIPEUS 5 µm 250×4.6 mm C18 column, water/acetonitrile = 90/10 to 40/60 over 20 min, flow rate = 0.600 mL/min, 1 = 214 nm) tR = 8.12 min. LRMS (ESI-QTOF) m/z: $[M+2H]^{2+}$ Calcd for $C_{60}H_{90}N_{16}O_{14}S_2$ 661.3; Found 661.7

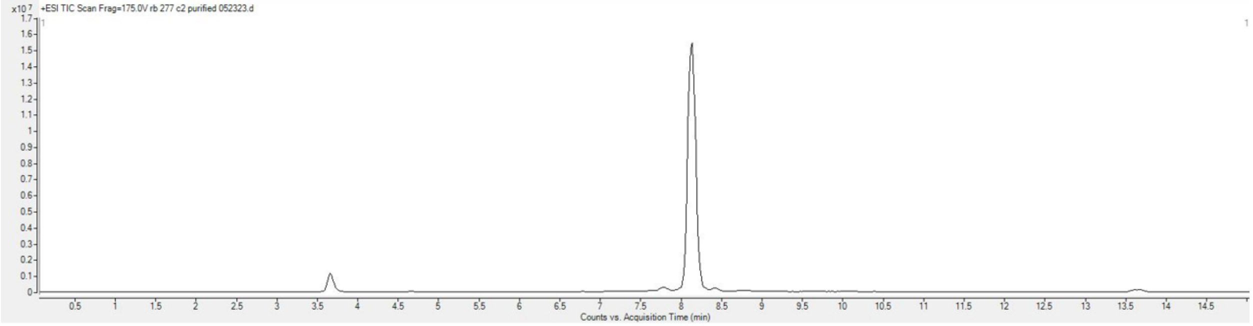

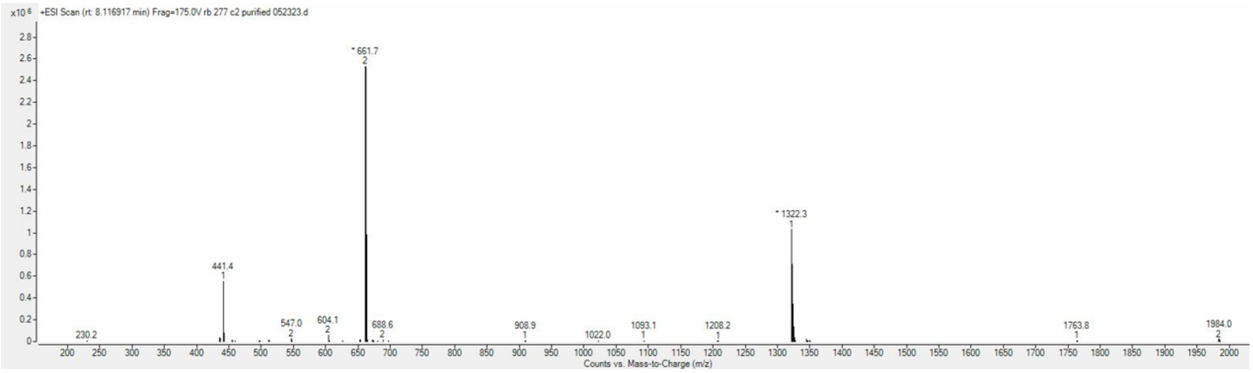

*Conjugation of **26** to **35***

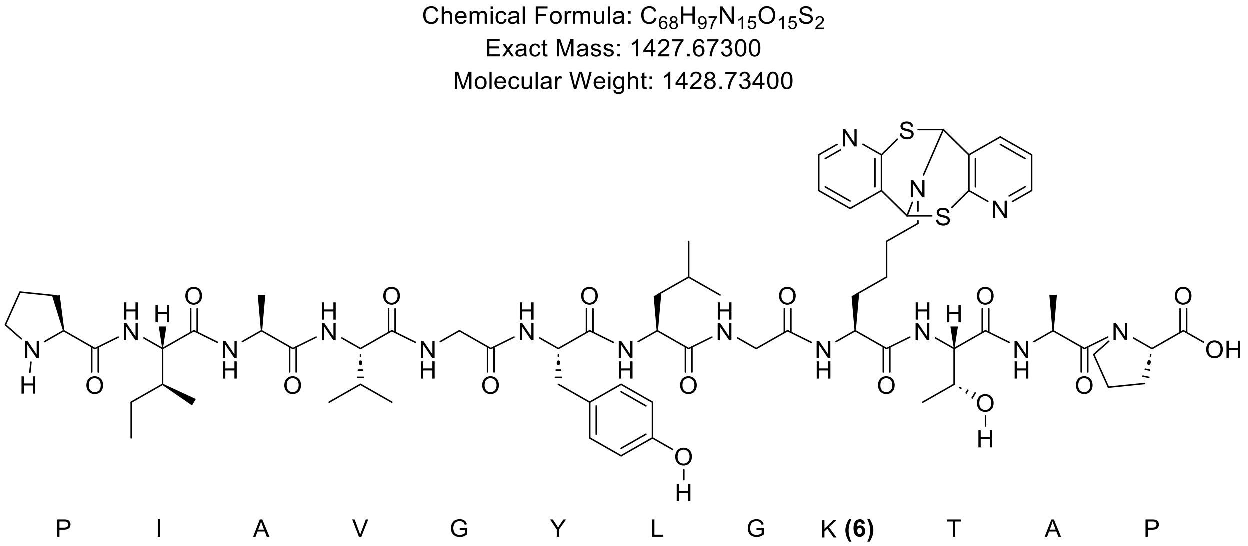

35

Peptide **26** was prepared by automated SPPS. 4.1 mg (0.0148 mmol) of **6** was dissolved in 104 µL of 0.5 M TCEP•HCl solution (0.0518 mmol) and was stirred at 38 °C for 10 minutes. 4.4 mg (0.0037 mmol, 12 mM) of **26** was dissolved in 200 µL of PBS buffer and administered directly into the solution. The reaction was adjusted to a pH of 6.3 and was stirred at 38 °C until bicyclic-formation was observed 27 hours later. Due to instability during preparative HPLC, the product was isolated using C2 reverse-phase flash column procedure described in **II. General Procedure**. Collected in twelve 6 mL fractions, isolated product was lyophilized to afford 2.1 mg (0.0015 mmol) of **35** as a flakey, white solid (42% yield). HPLC (Higgins Analytical CLIPEUS 5 µm 250×4.6 mm C18 column, water/acetonitrile = 90/10 to 40/60 over 20 min, flow rate = 0.600 mL/min, l = 214 nm) tR = 9.68 min. HRMS (ESI-QTOF) m/z: $[M+2H]^{2+}$ Calcd for $C_{68}H_{99}N_{15}O_{15}S_2$ 714.8438; Found 714.8464

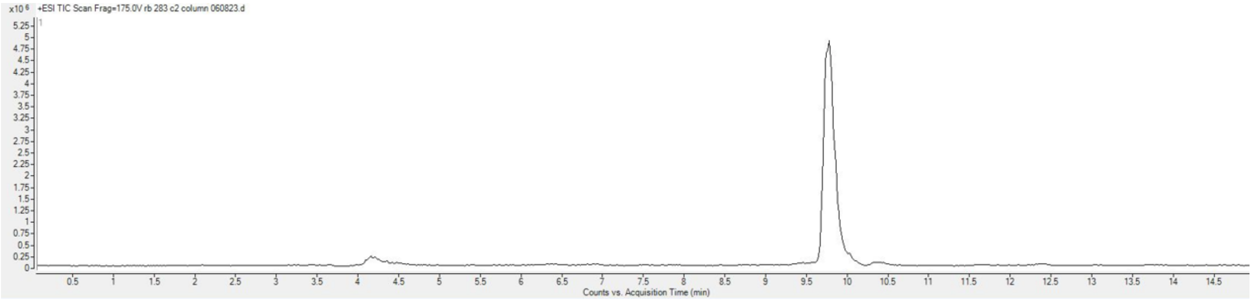
x10 6 +ESI TIC Scan Frag=175.0V rb 283 c2 column 060823.d
Counts vs. Acquisition Time (min)

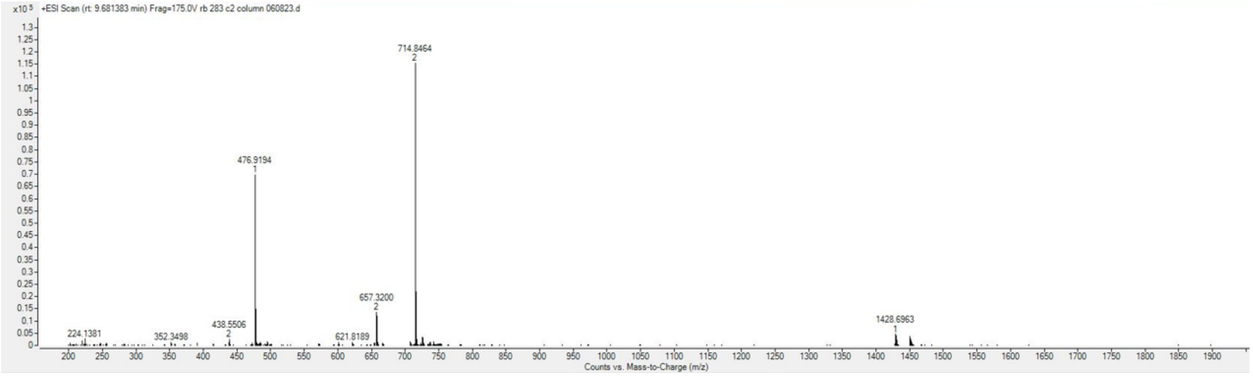
x10 5 +ESI Scan (rt: 9.681383 min) Frag=175.0V rb 283 c2 column 060823.d
714.8464
2
476.9194
1
657.3200
2
1428.6963
1
224.1381
352.3498
438.5506
2
621.8189
Counts vs. Mass-to-Charge (m/z)

VI. Investigation of Deconjugation Scope.

*Deconjugation of **14***

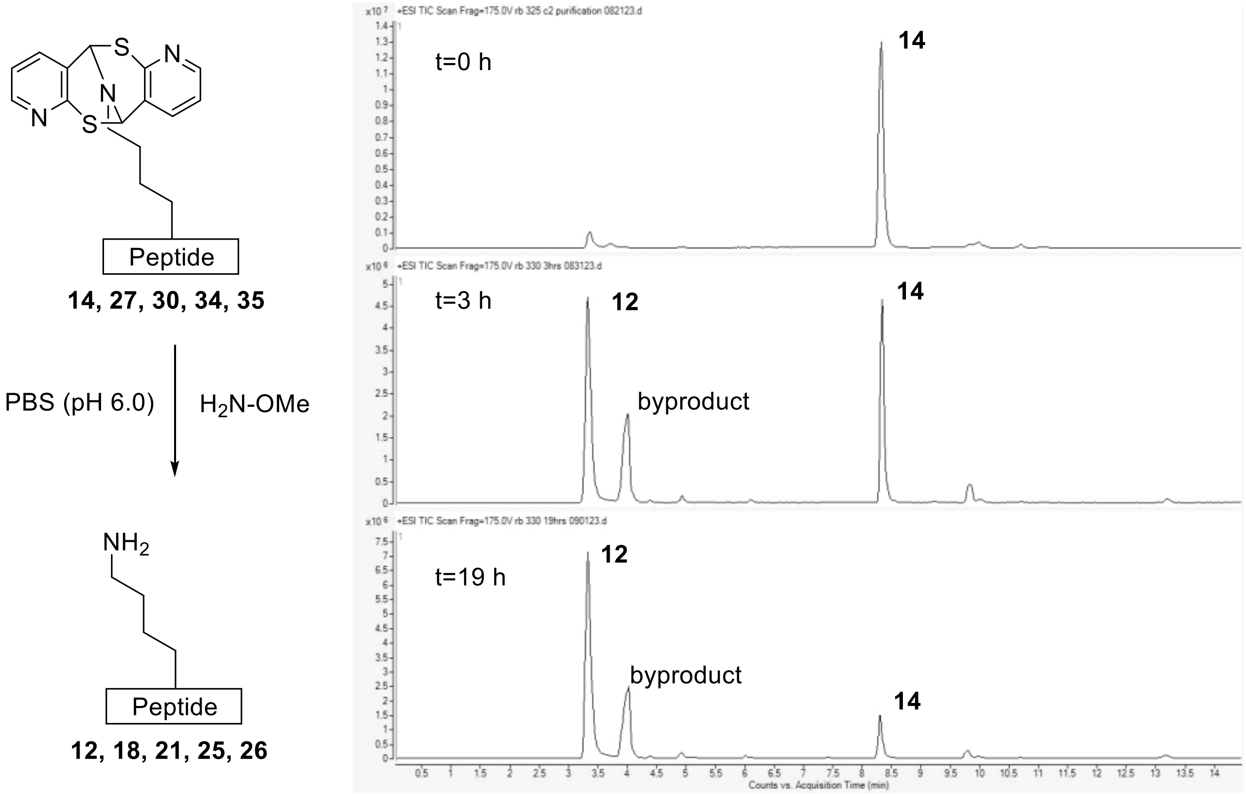

Raw LCMS spectrum of the deconjugation from peptide 14 at 0, 3, and 19hrs respectively.

Chemical Formula: $C_{63}H_{103}N_{21}O_{24}$
Exact Mass: 1537.74848
Molecular Weight: 1538.64000

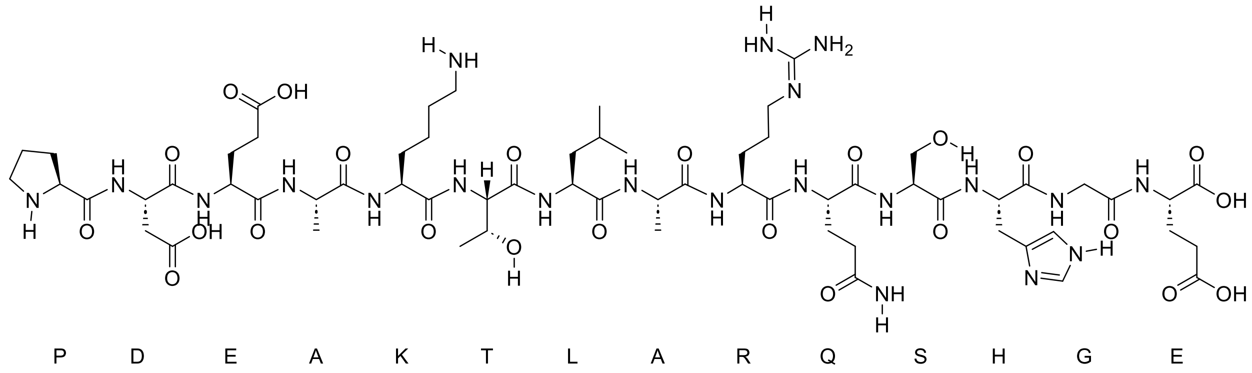

12

1.5 mg (0.0008 mmol) of conjugated peptide **14** was dissolved in a vial containing 160 µL of 26 mM methoxyamine•HCl in PBS buffer adjusted to pH 6.0. The reaction stirred at room temperature mixing at 360 rpm for 21 hours, monitored by LCMS. The conjugated peptide was reduced sharply. The initial peptide was recovered by preparative HPLC (10 to 70% solvent B over 20 min, Higgins Analytical CLIPEUS 5 µm 250×10 mm C18 column). Recovered peptide was lyophilized to afford 1.1 mg (0.0007 mmol) of **12** as a white powder (85 % yield). HPLC (Higgins Analytical CLIPEUS 5 µm 250×4.6 mm C18 column, water/acetonitrile = 90/10 to 40/60 over 20 min, flow rate = 0.600 mL/min, l = 214 nm) tR = 3.23 min. LRMS (ESI-QTOF) m/z: $[M+3H]^{3+}$ Calcd for $C_{63}H_{106}N_{21}O_{24}$ 513.6; found 513.5

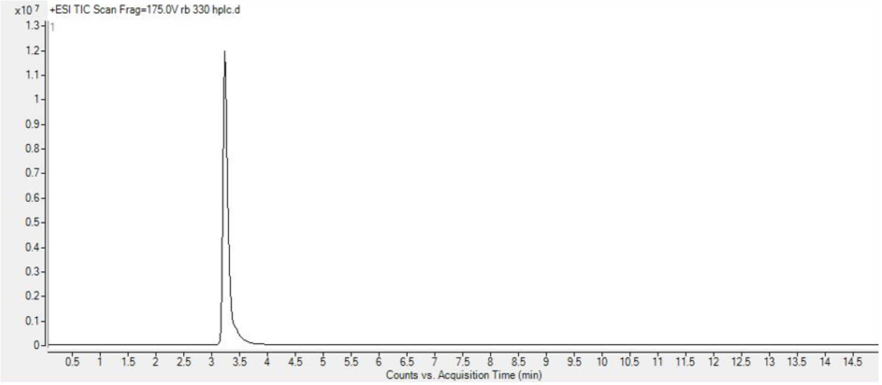

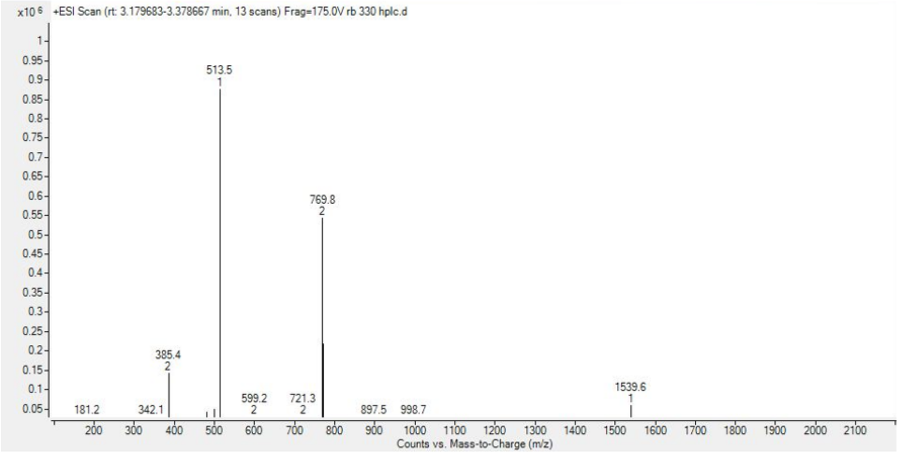

Deconjugation of ***27***

Chemical Formula: $C_{62}H_{106}N_{20}O_{20}$
Exact Mass: 1450.78923
Molecular Weight: 1451.65000

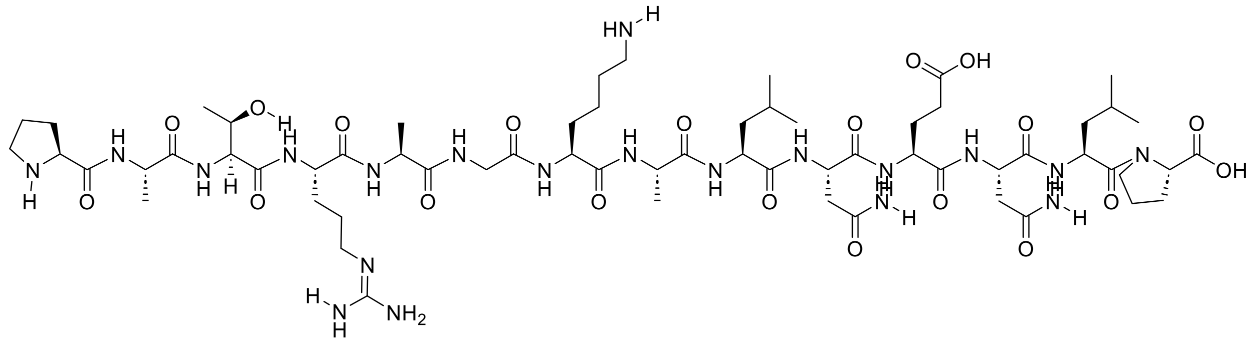

18

1 mg (0.0006 mmol) of conjugated peptide **27** was dissolved in a vial containing 200 µL of 26 mM methoxyamine•HCl in PBS buffer adjusted to pH 6.0. The reaction stirred at room temperature, mixing at 360 rpm for 6 hours, monitored by LCMS. The conjugated peptide was reduced sharply. The initial peptide was recovered by preparative HPLC (10 to 70% solvent B over 20 min, Higgins Analytical CLIPEUS 5 µm 250×10 mm C18 column). Recovered peptide was lyophilized to afford 0.81 mg (0.0005 mmol) of **18** as a white powder (93 % yield). HPLC (Higgins Analytical CLIPEUS 5 µm 250×4.6 mm C18 column, water/acetonitrile = 90/10 to 40/60 over 20 min, flow rate = 0.600 mL/min, l = 214 nm) tR = 3.52 min. LRMS (ESI-QTOF) m/z: $[M+3H]^{3+}$ Calcd for $C_{62}H_{109}N_{20}O_{20}$ 484.6; Found 484.6

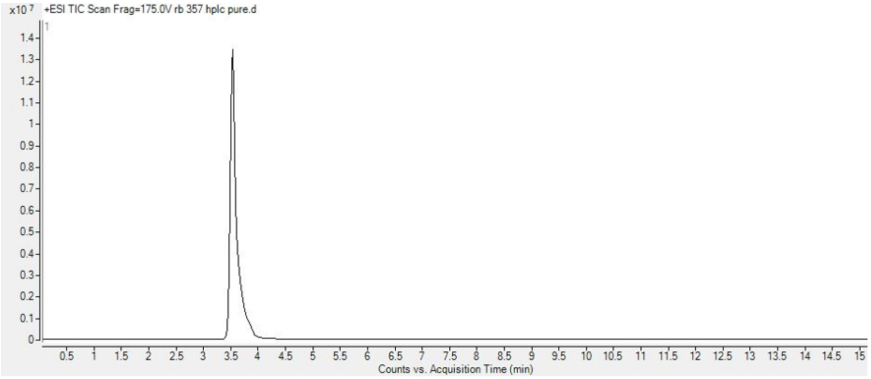

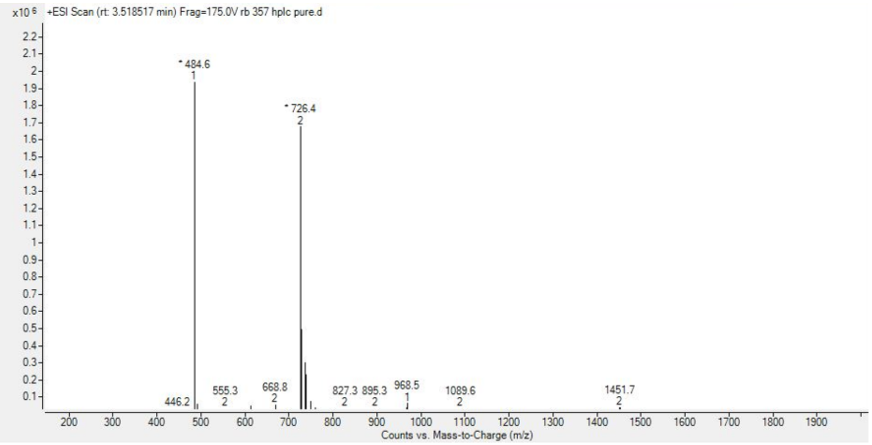

Deconjugation of ***30***

Chemical Formula: $C_{53}H_{96}N_{16}O_{16}$
Exact Mass: 1212.71902
Molecular Weight: 1213.44700

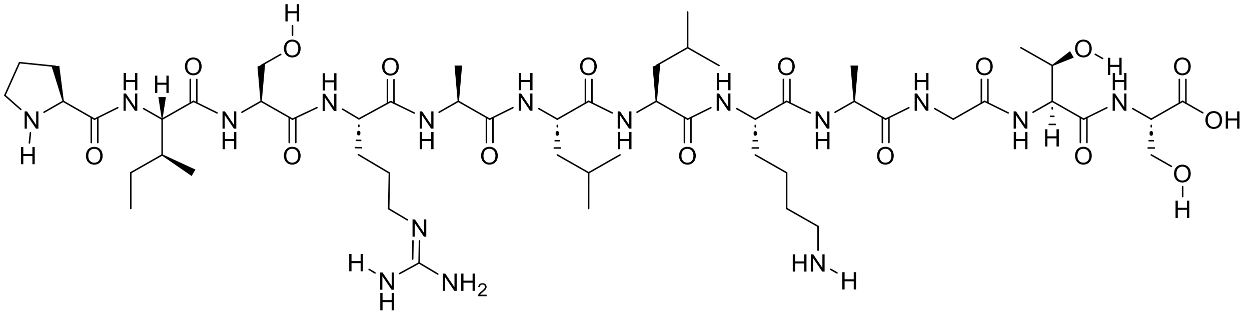

P I S R A L L K A G T S

21

0.7 mg (0.0005 mmol) of conjugated peptide **30** was dissolved in a vial containing 200 μL of 42 mM methoxyamine•HCl in PBS buffer adjusted to pH 6.0. The reaction stirred at 38 °C mixing at 360 rpm for 20 hours, monitored by LCMS. The conjugated peptide was reduced sharply. The initial peptide was

recovered by preparative HPLC (10 to 70% solvent B over 20 min, Higgins Analytical CLIPEUS 5 µm 250×10 mm C18 column). Recovered peptide was lyophilized to afford 0.32 mg (0.0003 mmol) of **21** as a white powder (55 % yield). HPLC (Higgins Analytical CLIPEUS 5 µm 250×4.6 mm C18 column, water/acetonitrile = 90/10 to 40/60 over 20 min, flow rate = 0.600 mL/min, l = 214 nm) tR = 4.20 min. LRMS (ESI-QTOF) m/z: $[M+3H]^{3+}$ Calcd for $C_{53}H_{99}N_{16}O_{16}$ 405.2; Found 405.2

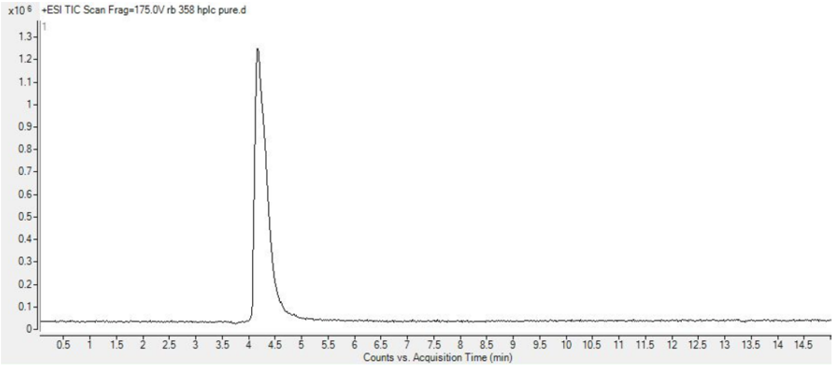

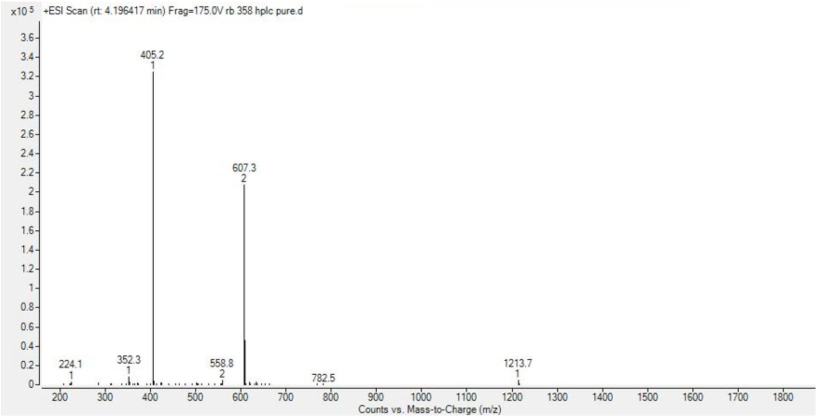

*Deconjugation of **34***

Chemical Formula: $C_{48}H_{82}N_{14}O_{14}$
Exact Mass: 1078.61349
Molecular Weight: 1079.26800

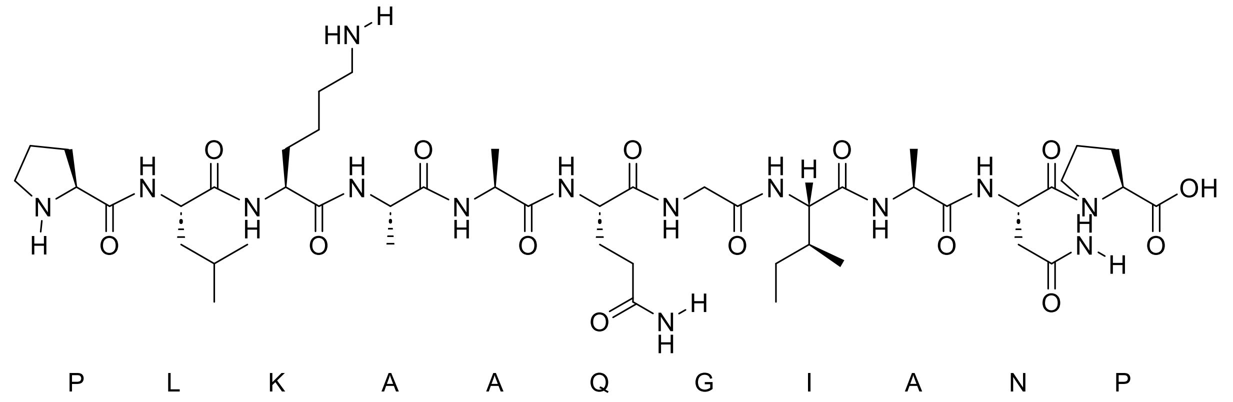

25

1 mg (0.0008 mmol) of conjugated peptide **34** was dissolved in a vial containing 200 µL of 76 mM methoxyamine•HCl in PBS buffer adjusted to pH 6.0. The reaction stirred at 38 °C mixing at 360 rpm for 21 hours, monitored by LCMS. The conjugated peptide was reduced sharply. The initial peptide was recovered by preparative HPLC (10 to 70% solvent B over 20 min, Higgins Analytical CLIPEUS 5 µm 250×10 mm C18 column). Recovered peptide was lyophilized to afford 0.77 mg (0.0007 mmol) of **25** as a white powder (95 % yield). HPLC (Higgins Analytical CLIPEUS 5 µm 250×4.6 mm C18 column, water/acetonitrile = 90/10 to 40/60 over 20 min, flow rate = 0.600 mL/min, l = 214 nm) tR = 3.43 min. LRMS (ESI-QTOF) m/z: $[M+H]^+$ Calcd for $C_{48}H_{83}N_{14}O_{14}$ 1079.6; Found 1079.7

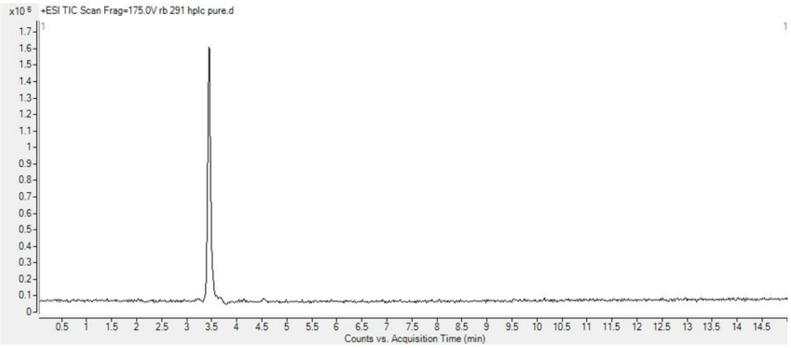

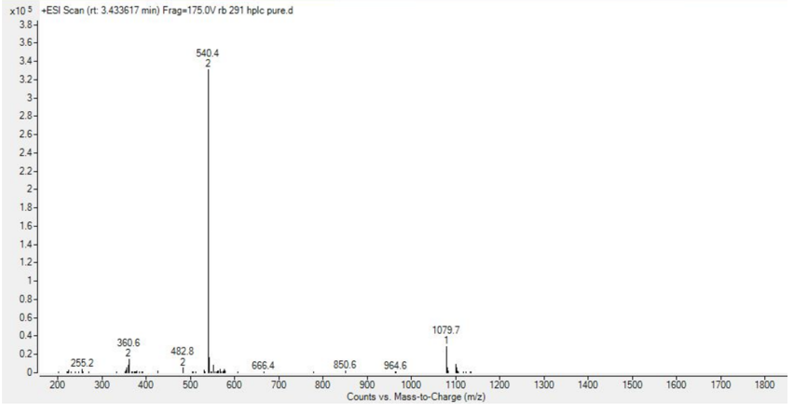

*Deconjugation of **35***

Chemical Formula: $C_{56}H_{91}N_{13}O_{15}$
Exact Mass: 1185.67576
Molecular Weight: 1186.42000

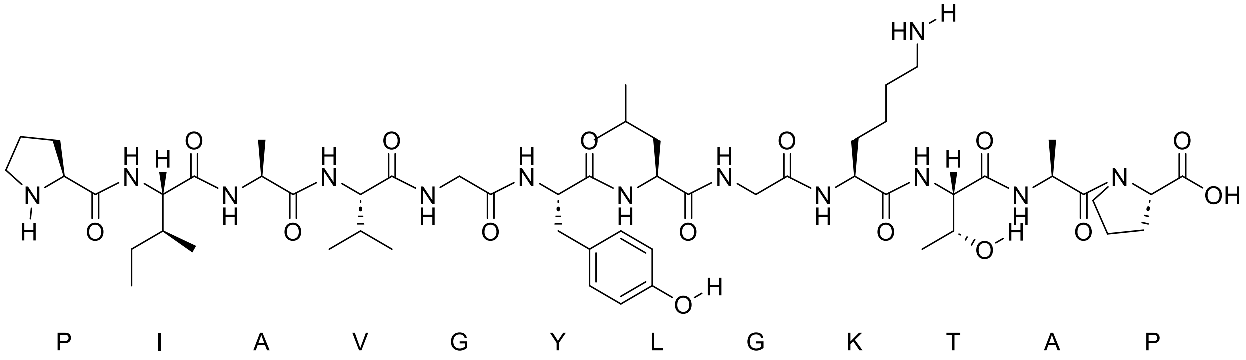

26

0.7 mg (0.0005 mmol) of conjugated peptide **35** was dissolved in a vial containing 200 µL of 67 mM methoxyamine•HCl in PBS buffer adjusted to pH 6.0. The reaction stirred at 38 °C mixing at 360 rpm for 6 hours, monitored by LCMS. The conjugated peptide was reduced sharply. The initial peptide was recovered by preparative HPLC (10 to 70% solvent B over 20 min, Higgins Analytical CLIPEUS 5 µm

250×10 mm C18 column). Recovered peptide was lyophilized to afford 0.5 mg (0.0004 mmol) of **26** as a white powder (86 % yield). HPLC (Higgins Analytical CLIPEUS 5 µm 250×4.6 mm C18 column, water/acetonitrile = 90/10 to 40/60 over 20 min, flow rate = 0.600 mL/min, l = 214 nm) tR = 11.08 min. LRMS (ESI-QTOF) m/z: $[M+H]^+$ Calcd for $C_{56}H_{92}N_{13}O_{15}$ 1186.7; Found 1186.7

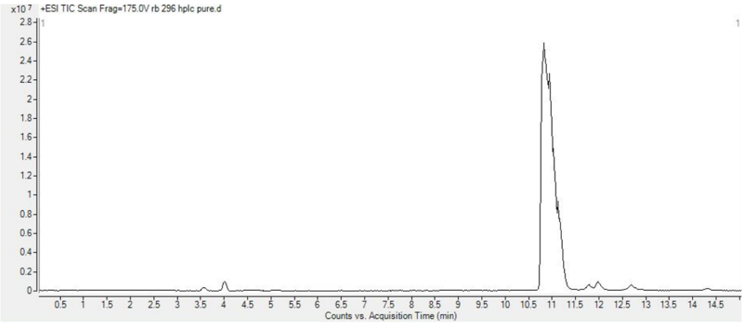

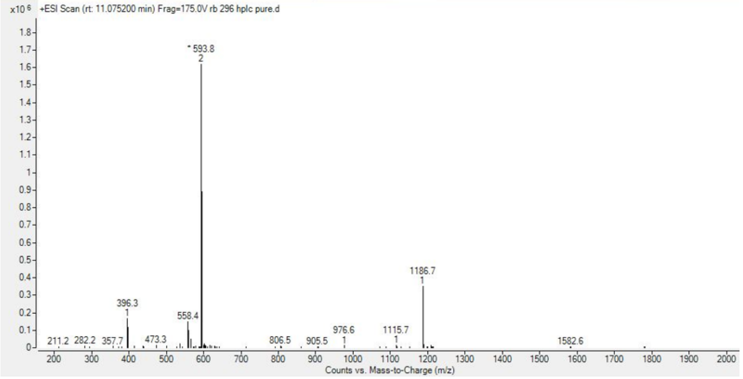

VII. Conjugation to Recombinant Insulin.

Liquid Chromatography-Mass Spectra by electrospray ionization (ESI) were obtained using an Agilent G6530BA Q-TOF Mass Spectrometer equipped with a Higgins Analytical PROTO 300 5 µm 250 ×4.6 mm C4 column. Flow rate followed 1.0 mL/min-0.8 mL/min with a gradient of 10%-60% Acetonitrile, 1% Formic acid (against H_2O, 1% Formic acid) for 20 minutes. HPLC was completed using the same procedure described in **II. General Procedure**.

*Conjugation of **6a** to Recombinant Insulin*

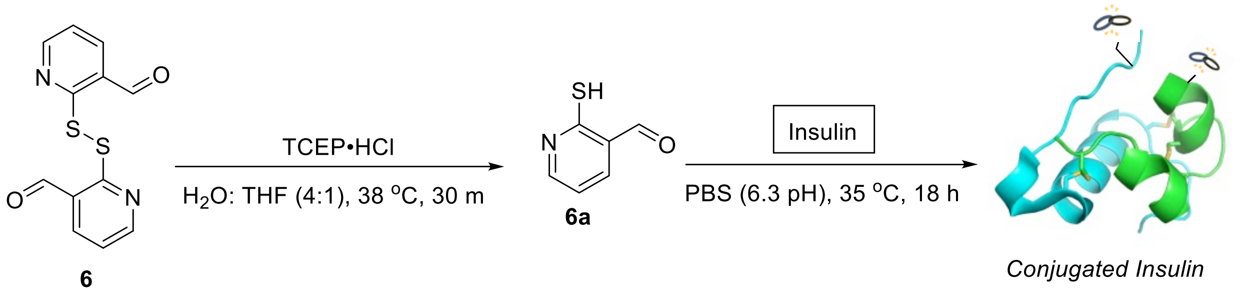

To a vial containing 10 mg of **6** (0.0362 mmol) was 400 µL of 0.5 M TCEP•HCl in H_2O: THF (4:1) added and stirred at 38 °C for 30 minutes. The monomer **6a** was extracted from the solution using ethyl acetate and water, and the solution was dried with Na_2SO_4 before concentrating via vacuo. All of the afforded mercaptan-monomer **6a** was then dispensed into a clean vial, and 300 µL of degassed PBS: THF (4:1) solution containing 2 mg (0.0003 mmol, 1 mM) of human insulin recombinant was added. The solution was adjusted to a pH of 6.3, and was stirred at 35 °C for 18 hours, monitored by LCMS. Starting insulin was converted completely, affording two equivalent peaks referring to the doubly conjugated protein. The solution was filtrated through a C8 reverse-phase plug with a H_2O: MeCN (8:1) mixture to remove buffer salts and lyophilized to afford 1.5 mg (0.0002 mmol) of the raw powder.

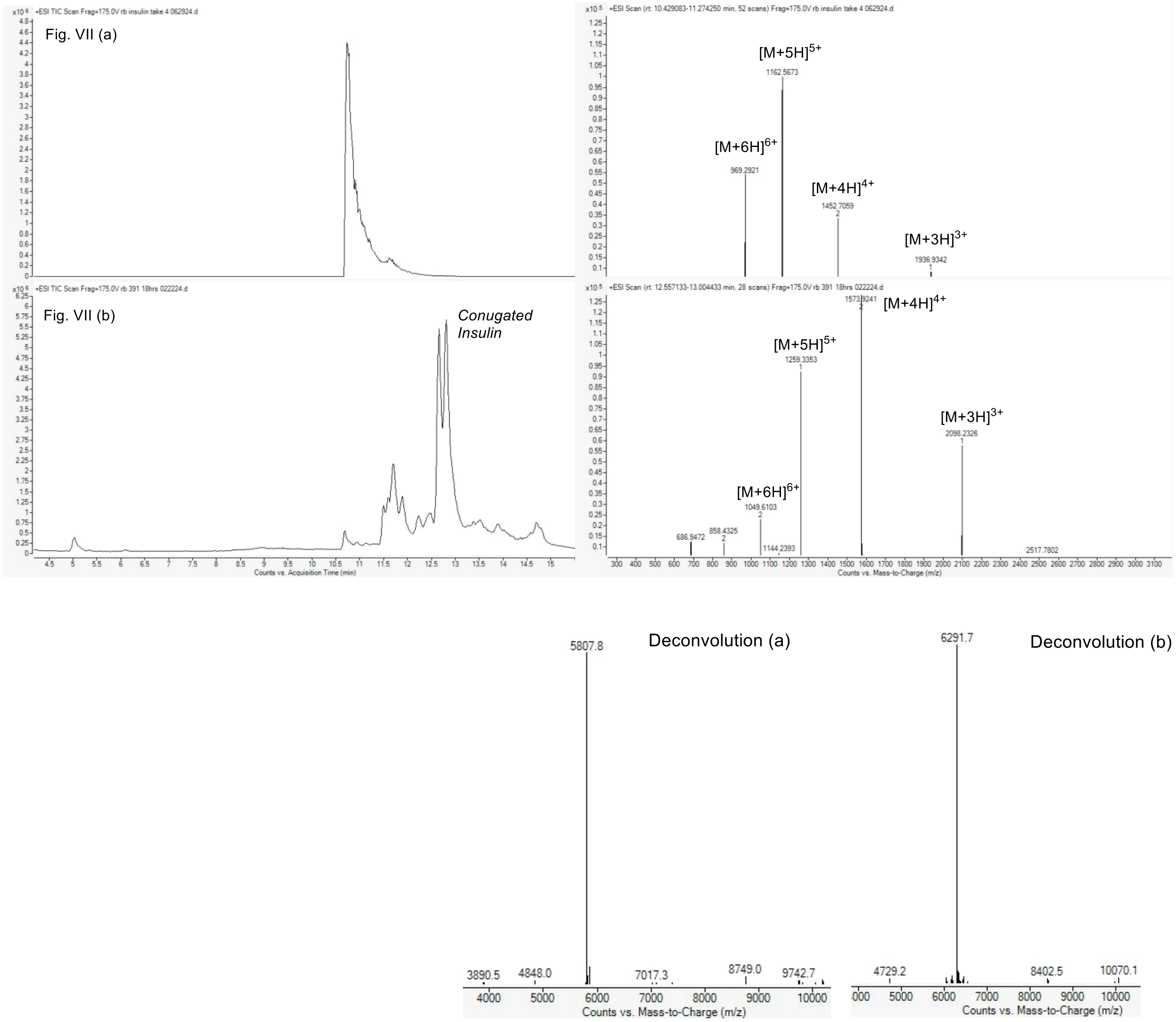

Fig. VII (a): LCMS spectrum of commercial recombinant insulin and its mass fragments. HPLC (Higgins Analytical Proto 5 µm 250×4.6 mm C4 column, water/acetonitrile = 90/10 to 40/60 over 20 min, flow rate = 1.000 mL/min to 0.800 mL/min over 20 min, l = 214 nm) tR = 10.73 min. LRMS (ESI-QTOF) m/z: Calcd for $C_{257}H_{383}N_{65}O_{77}S_{6}$ 5807.3; Found 5807.8

Fig VII (b): Raw LCMS spectrum for the conjugation of **6a** to insulin after 18 hours. Fragments associated with dual peaks of doubly-conjugated insulin. HPLC (Higgins Analytical Proto 5 µm 250×4.6 mm C4 column, water/acetonitrile = 90/10 to 40/60 over 20 min, flow rate = 1.000 mL/min to 0.800 mL/min over 20 min, l = 214 nm) tR = 12.66 min, 12.81 min. LRMS (ESI-QTOF) m/z: Calcd for $C_{281}H_{395}N_{69}O_{77}S_{10}$ 6292.3; Found 6291.7

Tandem mass study was performed with an Agilent G6530BA Q-TOF Mass Spectrometer equipped with a Higgins Analytical PROTO 300 5 µm 250 ×4.6 mm C4 column. Flow rate followed 0.8 mL/min with a gradient of 10%-60% Acetonitrile, 1% Formic acid (against H_2O, 1% Formic acid) for 20 minutes. Collision energy was set to 90 locked to base ion $[M+3H]^{3+}$: 2098.2317 m/z. Searching for fragments not involved in disulfide bonding, the results indicate conjugation to the lysine and n-terminus of the A-chain.

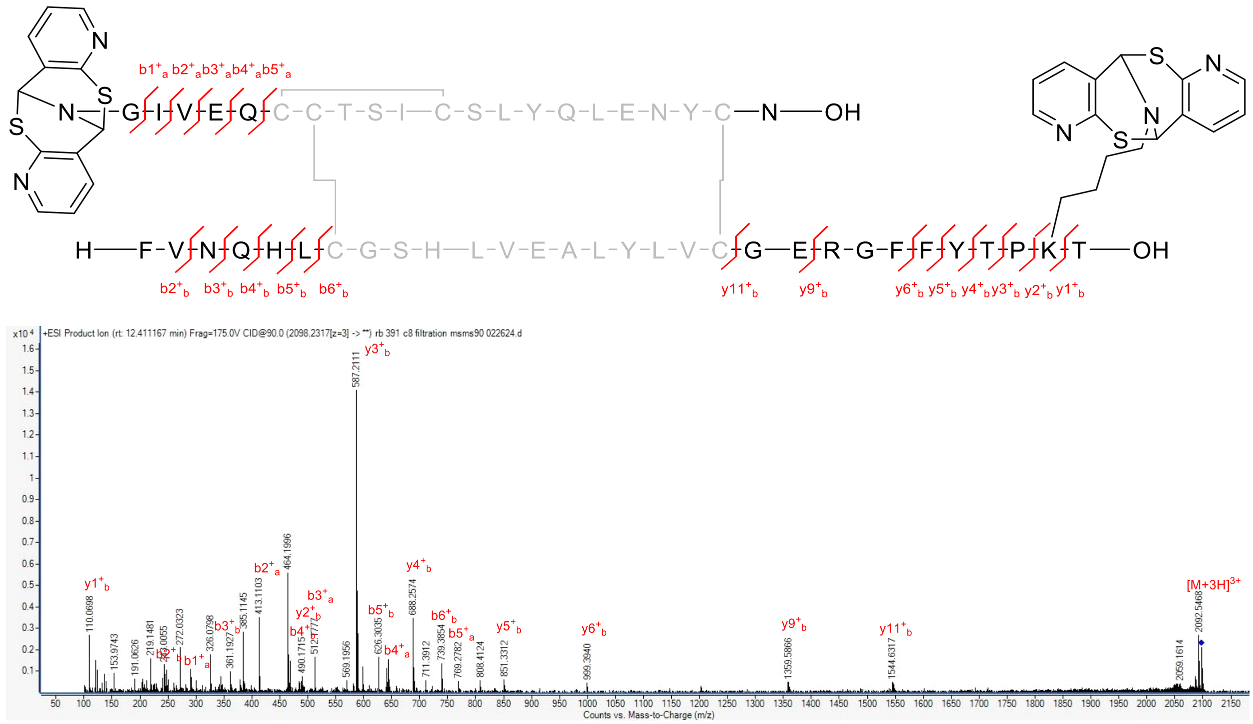

b⁺ ion	Calculated Mass Fragment (m/z)	Observed Mass Fragment (m/z)
$b1^{+}_{a}$	300.0288	300.0252
$b2^{+}_{a}$	413.1129	413.1103
$b3^{+}_{a}$	512.1813	512.1777
$b4^{+}_{a}$	641.2239	641.2328
$b5^{+}_{a}$	769.2824	769.2782

Table VII (a) Expected tandem mass fragmentations for the conjugation to the n-terminus of the A chain. 5/5 found.

y^+ ion	Calculated Mass Fragment (m/z)	Observed Mass Fragment (m/z)
$y1^+_b$	120.0656	120.0823
$y2^+_b$	490.1605	490.1715
$y3^+_b$	587.2133	587.2111
$y4^+_b$	688.2610	688.2574
$y5^+_b$	851.3243	851.3312
$y6^+_b$	998.3927	998.3897
$y7^+_b$	1145.4610	*not observed*
$y8^+_b$	1202.4830	*not observed*
$y9^+_b$	1358.5840	1358.5876
$y10^+_b$	1487.6260	*not observed*
$y11^+_b$	1544.6480	1544.6317

Table VII (b) Expected tandem mass fragmentations for the conjugation to the lysine residue of the B chain. 8/11 found.

b^+ ion	Calculated Mass Fragment (m/z)	Observed Mass Fragment (m/z)
$b1^+_b$	148.0757	*not observed*
$b2^+_b$	247.1442	247.1466
$b3^+_b$	361.1871	361.1927
$b4^+_b$	489.2457	489.2484
$b5^+_b$	626.3046	626.3035
$b6^+_b$	739.3886	739.3854

Table VII (c) Expected tandem mass fragmentations for free n-terminus of the B chain. 5/6 found.

VIII. Deconjugation from Insulin.

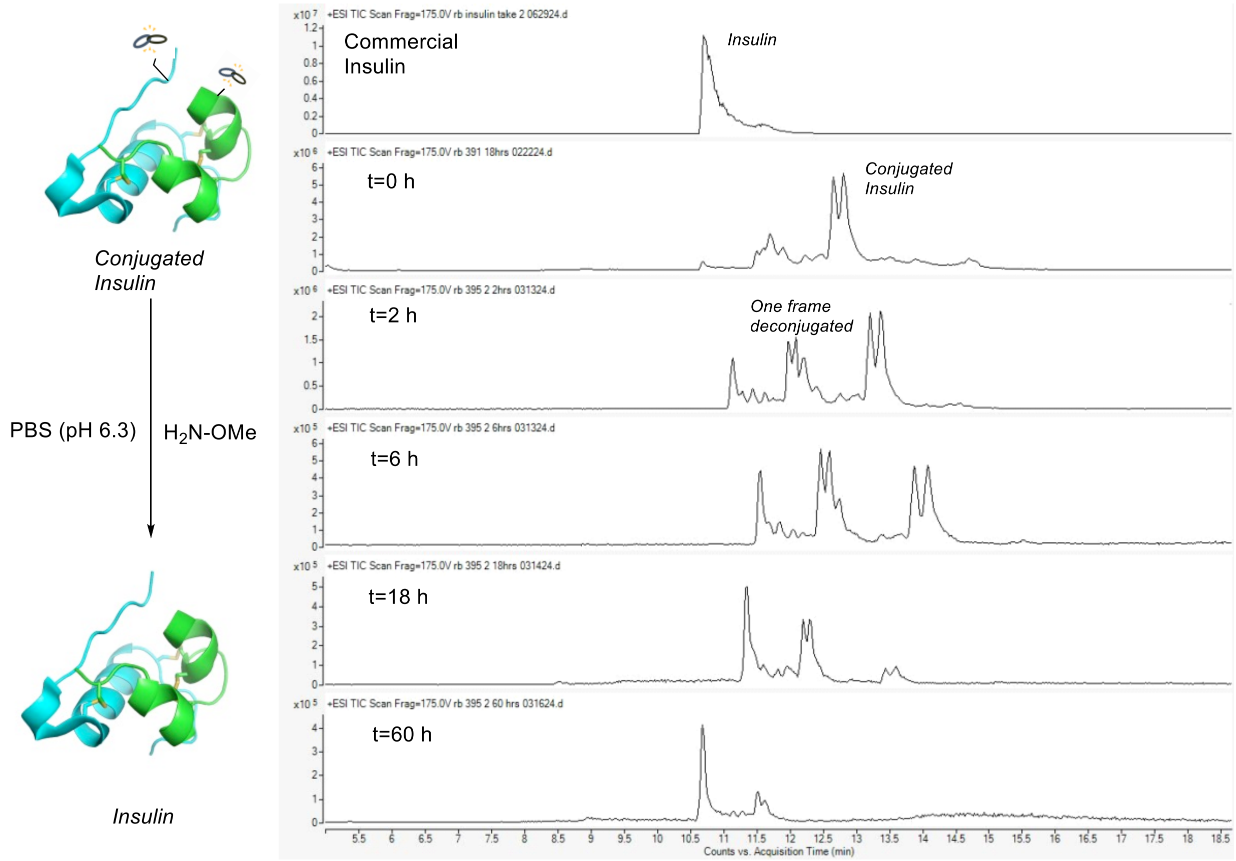

Raw LCMS spectrum of the deconjugation from insulin at 2, 6, 18, and 60hrs respectively.

0.9 mg (0.0001 mmol) of raw conjugated insulin powder was dissolved in a vial containing 300 µL of 7 mM methoxyamine•HCl in PBS: THF (4:1) solution adjusted to pH 6.3. The reaction stirred at room temperature mixing at 360 rpm for 60 hours, monitored by LCMS. The conjugated insulin peaks were reduced sharply. The initial protein was recovered by preparative HPLC (10 to 70% solvent B over 20 min, Higgins Analytical CLIPEUS 5 µm 250×10 mm C18 column). Recovered protein was lyophilized to afford 0.65 mg (0.0001 mmol) of free insulin (78 % yield). HPLC (Higgins Analytical Proto 5 µm 250×4.6 mm C4 column, water/acetonitrile = 90/10 to 40/60 over 20 min, flow rate = 1.000 mL/min to 0.800 mL/min over 20 min, l = 214 nm) tR = 10.21. LRMS (ESI-QTOF) m/z: Calcd for $C_{257}H_{383}N_{65}O_{77}S_6$ 5807.3; Found 5807.1

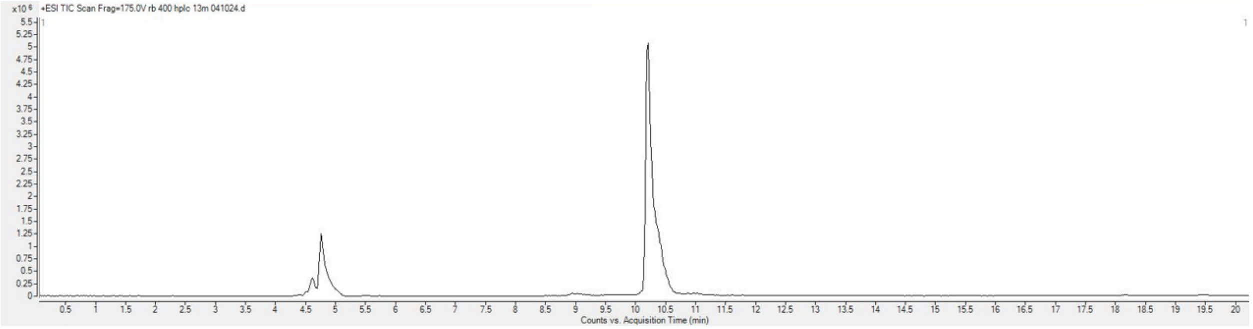
+ESI TIC Scan Frag=175.0V rb 400 hplc 13m 041024.d
Counts vs. Acquisition Time (min)

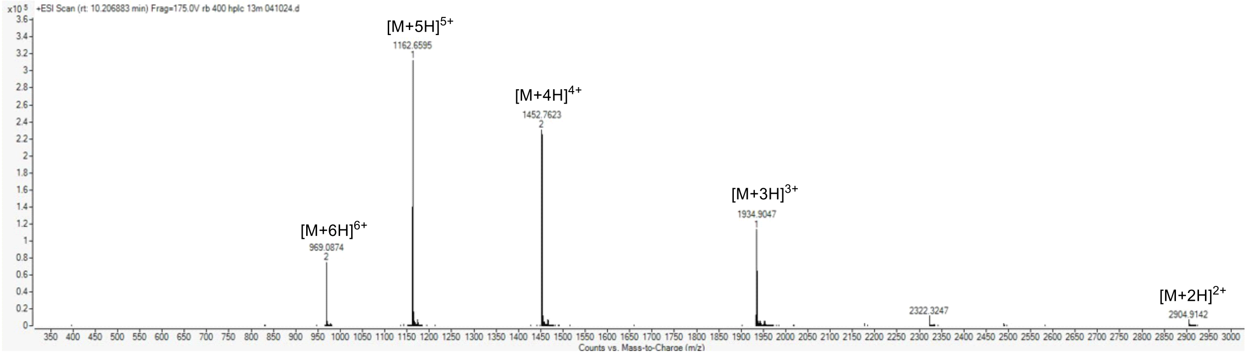
+ESI Scan (rt: 10.206883 min) Frag=175.0V rb 400 hplc 13m 041024.d
[M+5H]5+
1162.6595
[M+4H]4+
1452.7623
[M+3H]3+
1934.9047
[M+6H]6+
969.0874
2322.3247
[M+2H]2+
2904.9142
Counts vs. Mass-to-Charge (m/z)

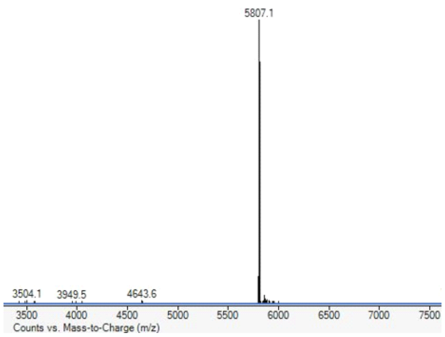
Deconvoluted
5807.1
3504.1
3949.5
4643.6
Counts vs. Mass-to-Charge (m/z)

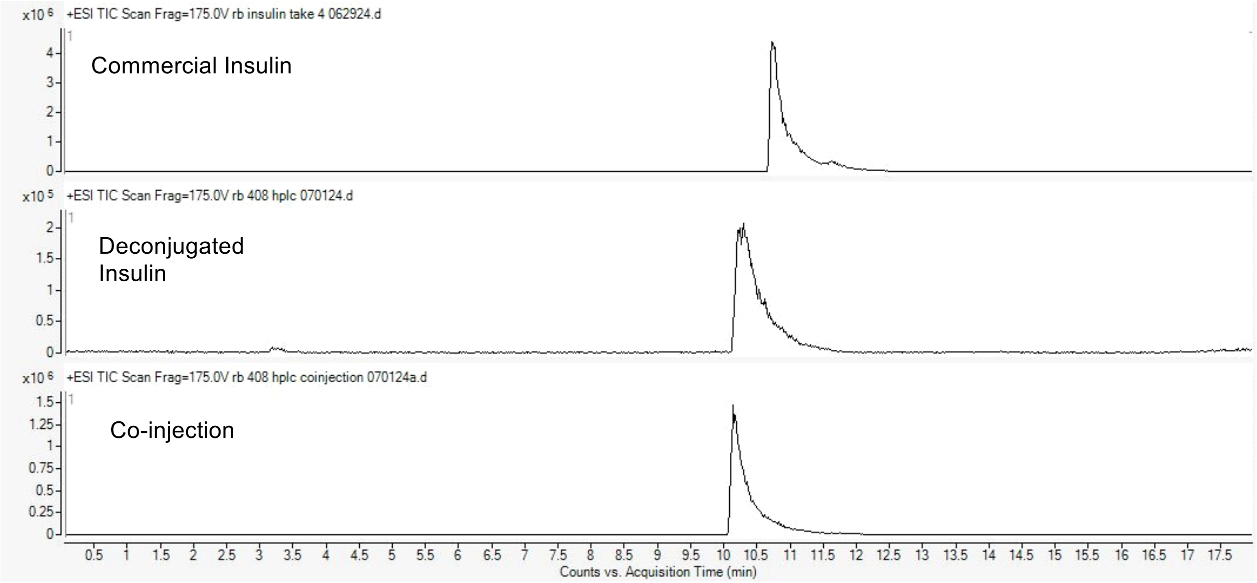
+ESI TIC Scan Frag=175.0V rb insulin take 4 062924.d
Commercial Insulin
+ESI TIC Scan Frag=175.0V rb 408 hplc 070124.d
Deconjugated
Insulin
+ESI TIC Scan Frag=175.0V rb 408 hplc coinjection 070124a.d
Co-injection
Counts vs. Acquisition Time (min)

IX. NMR Spectra.

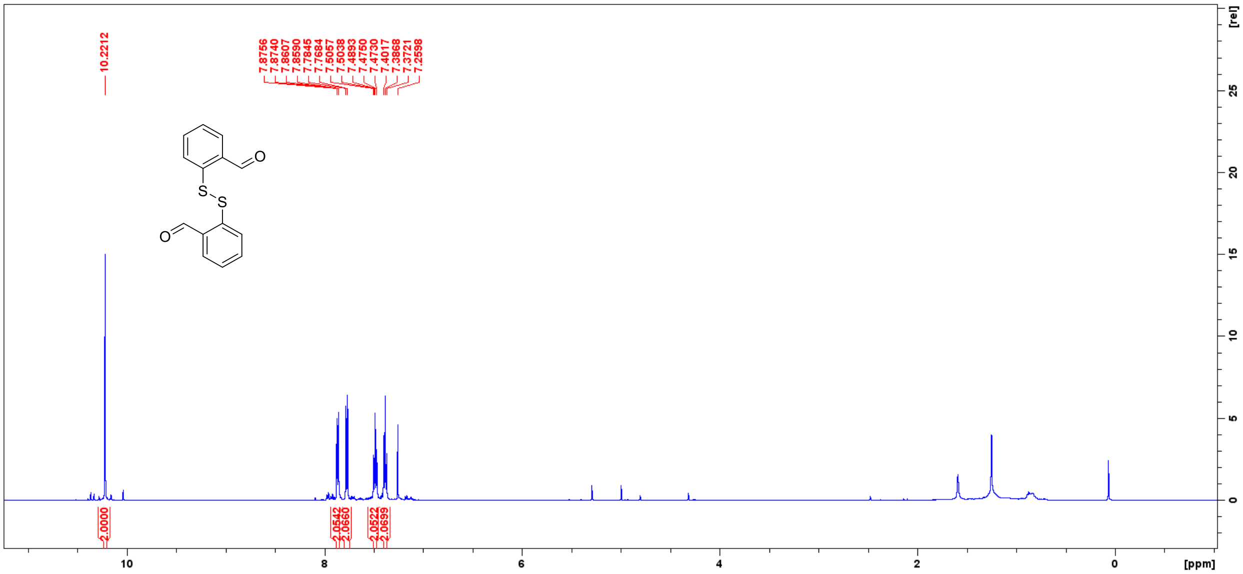

^{1}H NMR Spectrum of **3** in $CDCl_3$-d (500 MHz)

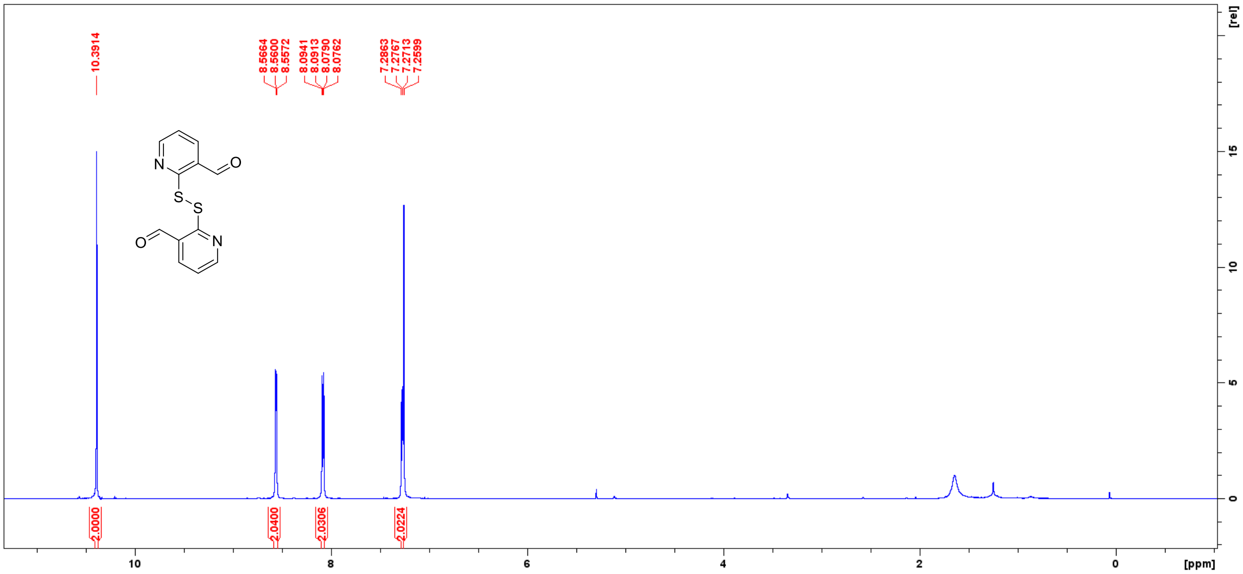

^{1}H NMR Spectrum of **6** in $CDCl_3$-d (500 MHz)

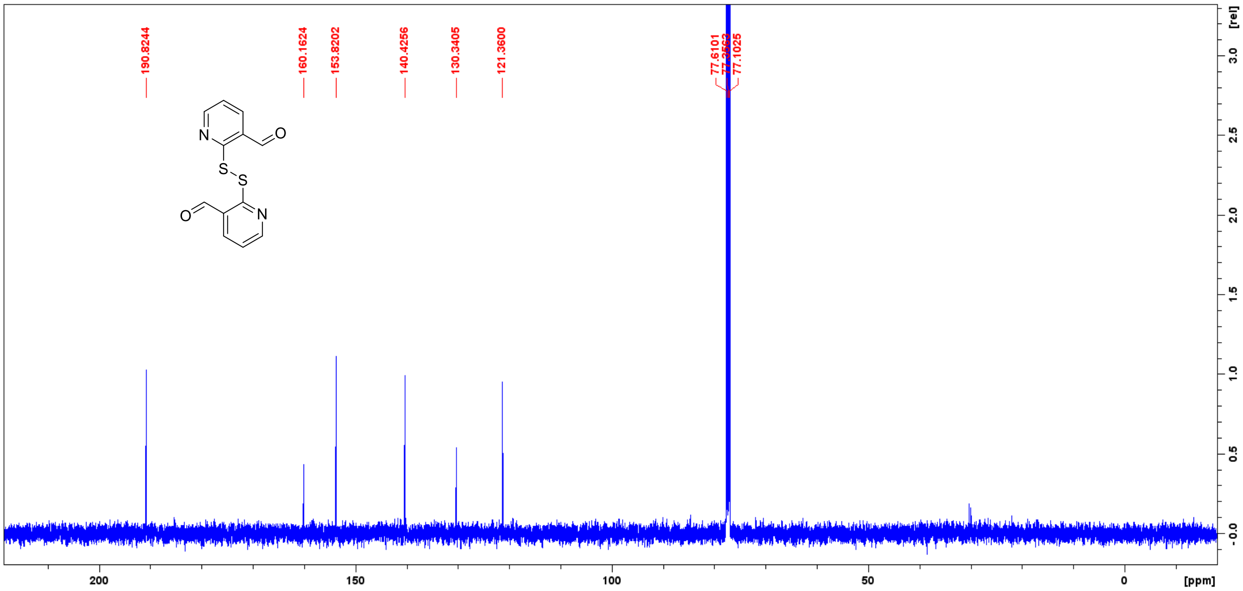

^{13}C NMR Spectrum of **6** in $CDCl_3$-d (125 MHz)

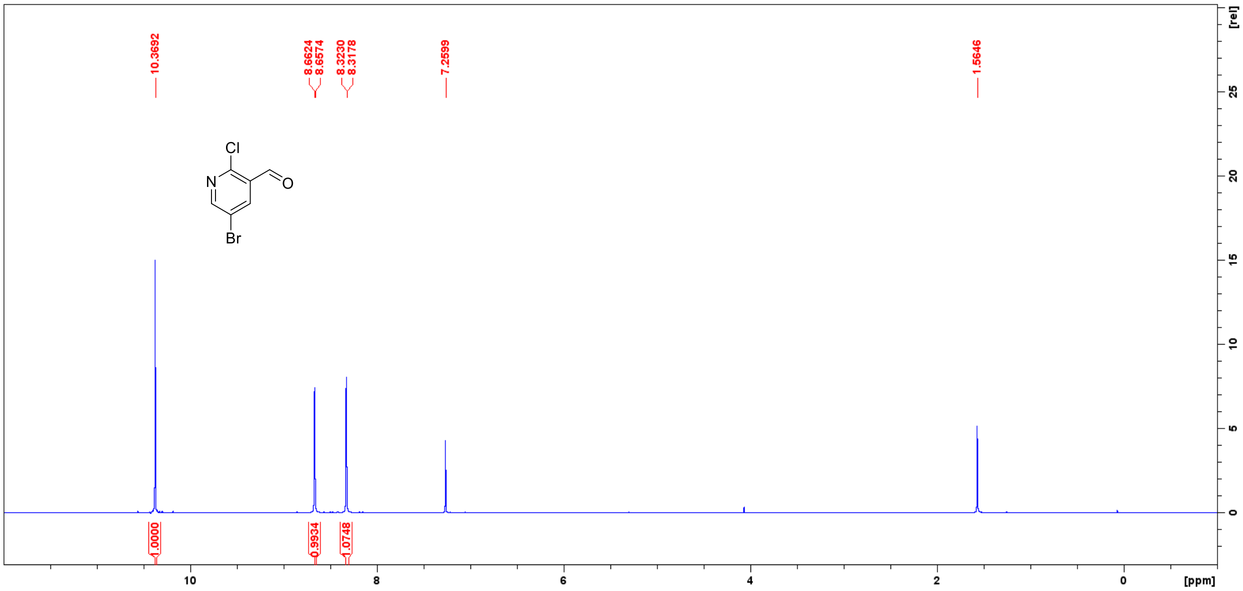

^{1}H NMR Spectrum of **8** in $CDCl_3$-d (500 MHz)

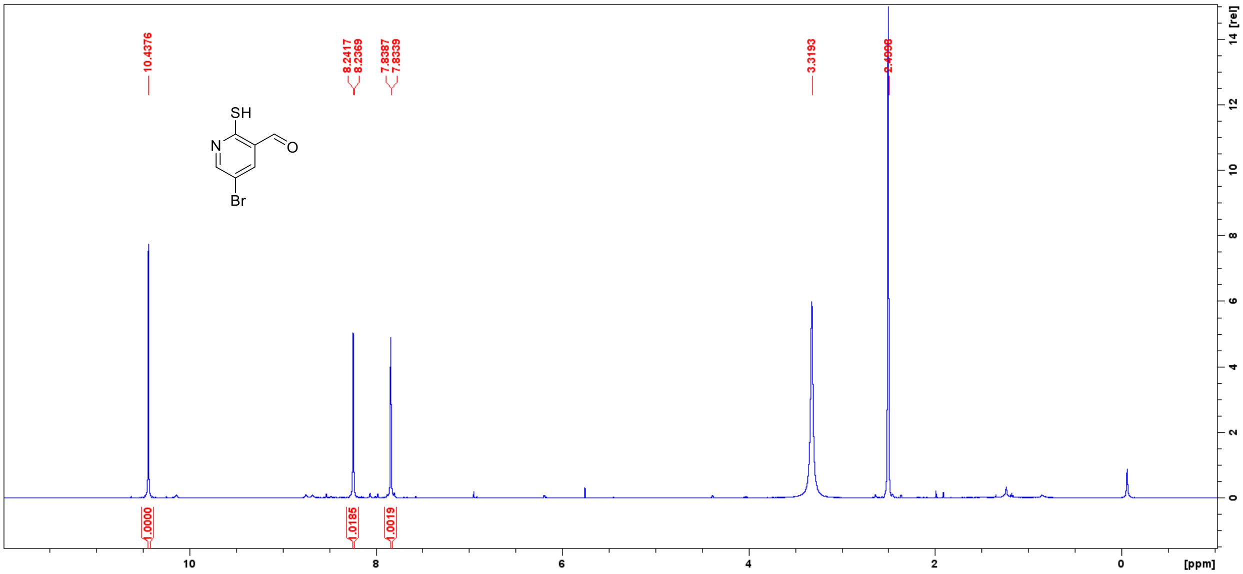

^{1}H NMR Spectrum of **9** in DMSO-d6 (500 MHz)

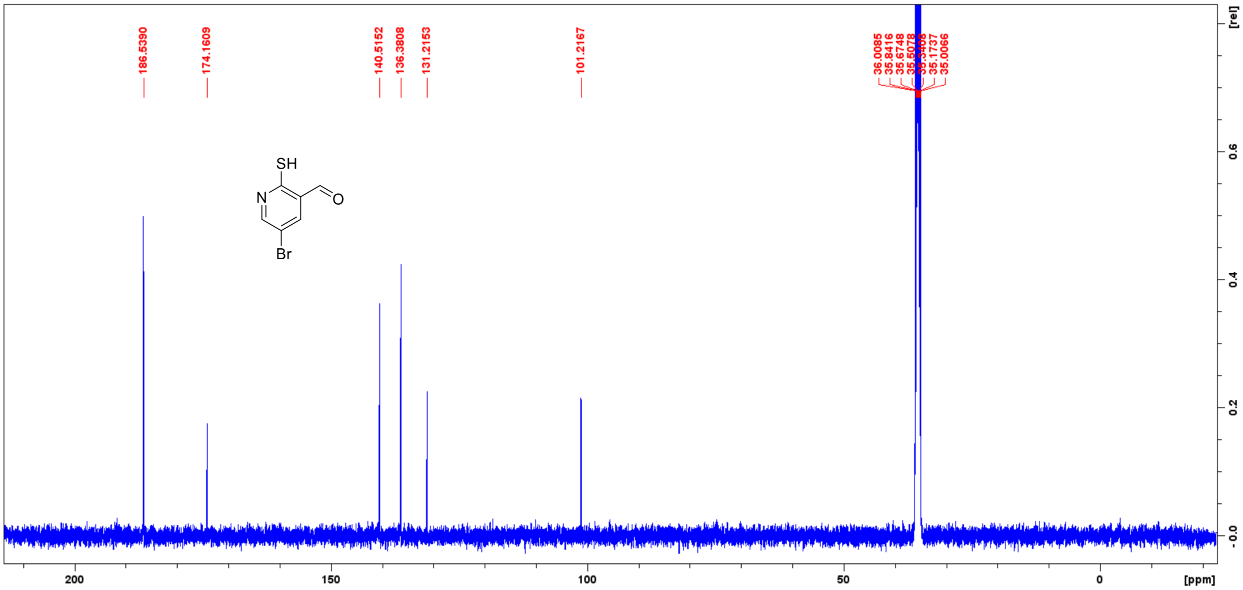

^{13}C NMR Spectrum of **9** in DMSO-d6 (125 MHz)

X. References.

(1) Raj. M.; Wu, H.; Blosser, S. L.; Vittoria, M. A.; Arora, P. S. Aldehyde Capture Ligation for Synthesis of Native Peptide Bonds. *J. Am. Chem. Soc*. **2015,** *137* (21)**,** 6932–6940.
